# Seasonal catchment memory of high mountain rivers in the Tibetan Plateau

**DOI:** 10.1038/s41467-023-38966-9

**Published:** 2023-06-01

**Authors:** Haiting Gu, Yue-Ping Xu, Li Liu, Jingkai Xie, Lu Wang, Suli Pan, Yuxue Guo

**Affiliations:** Institute of Water Science and Engineering, College of Civil Engineering and Architecture, Zhejiang University, 310058 Hangzhou, China

**Keywords:** Hydrology, Hydrology

## Abstract

Rivers originating in the Tibetan Plateau are crucial to the population in Asia. However, research about quantifying seasonal catchment memory of these rivers is still limited. Here, we propose a model able to accurately estimate terrestrial water storage change (TWSC), and characterize catchment memory processes and durations using the memory curve and the influence/domination time, respectively. By investigating eight representative basins of the region, we find that the seasonal catchment memory in precipitation-dominated basins is mainly controlled by precipitation, and that in non-precipitation-dominated basins is strongly influenced by temperature. We further uncover that in precipitation-dominated basins, longer influence time corresponds to longer domination time, with the influence/domination time of approximately six/four months during monsoon season. In addition, the long-term catchment memory is observed in non-precipitation-dominated basins. Quantifying catchment memory can identify efficient lead times for seasonal streamflow forecasts and water resource management.

## Abstract (web summary)

This study investigates the seasonal catchment memory of rivers in the Tibetan Plateau, with the help of a water storage change model. Understanding catchment memory can aid in hydrological forecasts and water resource management

## Introduction

The Tibetan Plateau, known as the roof of the world, is the source of many major rivers^1^. These rivers nurture people in East Asia, South Asia, and Southeast Asia, where about half of the world’s population lives^2^. However, with the changing frequency and severity of hydrological extremes under climate change, the risks of water disasters, such as floods and droughts, are increasing in the Tibetan Plateau^3^. Therefore, it is crucial to study seasonal hydrological forecasts to manage water resources and ensure food security in the wide regions of the Tibetan Plateau. Seasonal forecast refers to the prediction of land surface hydrologic variables at sub-seasonal to seasonal timescales and the forecasting of persistent land surface hydrologic anomalies^4^. The concept of seasonal catchment memory, which extends the lead times and predictability of streamflow compared to rainfall forecasts^5^, is often used in seasonal forecasts. To improve the skill of seasonal forecasts and water resource management, it is necessary to understand the response mechanism of a catchment to incoming precipitation^6^.

Incoming precipitation can be retained in various forms, such as soil water, snow and ice, lake water, groundwater etc., and can subsequently be released as streamflow and evapotranspiration (ET). The storage of water can impact a catchment’s response to subsequent precipitation, as if the catchment area possesses a memory and can memorize past information^7^. Catchment memory can be categorized into two types based on the duration, namely multiyear (long-term) memory and seasonal (short-term) memory^8^. The former is usually analyzed on an annual time step^9^, and the latter on a monthly or daily time step^10^. The lagged correlation is the most commonly used method for describing catchment memory, such as Pearson’s correlation between selected river flow signatures and the average river flow in antecedent months^11^, and Spearman’s correlation between groundwater storage, precipitation and runoff^12^. The lagged correlation method is straightforward to use and understand, focusing mainly on exploring correlations between different hydrologic variables, but is unable to establish a function between them. Recently, alternative methods have been proposed for studying catchment memory. A popular way in hydrological forecasting is to calculate the potential parameter for water retention (or memory capacity) and the rate of water release, such as the baseflow index and the groundwater recession coefficient, as discussed in the study of Sutanto and Van Lanen^13^. However, this approach primarily focuses on the catchment state to assist hydrological forecasting, with little consideration of the catchment memory process and its duration. Hysteresis loops between hydrologic variables can visualize catchment memory and are typically used for qualitative analysis of catchment memory^14^, which provides a tool for analyzing the relationships among precipitation, streamflow, and water storage. In the study of de Lavenne et al.^8^, the Gamma distribution-based catchment forgetting curve was proposed to quantify multiyear catchment memories via precipitation, streamflow, and ET. This method describes how the catchment memory fades over time (i.e., the catchment memory process) rather than providing a single value of catchment memory like other methods, but at the same time it neglects the assessment of memory duration. Generally, for seasonal catchment memory, there is currently a lack of a well-defined method for characterizing its process and duration.

Catchment memory is typically associated with water storage, which is often simulated using hydrological models in previous studies^12,15,16^. Since 2002, the Gravity and Climate Recovery Experiment (GRACE) satellite and its Follow-on (GRACE-FO) satellite have monitored terrestrial water storage (TWS), which includes land surface hydrological fluxes such as streamflow, soil moisture, and groundwater^17,18^. Like in other fields of hydrology, the GRACE TWS anomalies (TWSA) data is used as an observed water storage dataset in the study of catchment memory^19^. For instance, Opie et al. used the correlation between the GRACE-derived changes in groundwater storage and precipitation to describe the long-term and short-term catchment memory^20^; Xie et al. employed GRACE TWSA data to calculate the Flood Potential Index^21^, which describes memory capacity. Compared to model-derived TWS data, GRACE TWS data can provide more accurate and direct measurements of TWS without relying on the assumption that changes in TWS are insignificant on annual and longer timescales^22^. Previous studies have demonstrated the applicability of GRACE data to the Tibetan Plateau^23,24^. Li et al. found that that JPL-M likely provides reliable TWS estimates for the Tibetan Plateau^25^.

Based on the above literature, it is evident that two issues about seasonal catchment memory still need to be addressed. First, there is a need to disentangle the linkage among precipitation, streamflow and TWS to understand how catchments memorize precipitation. Second, there is a need to quantify seasonal catchment memory with both process and duration.

In this work, we focus on investigating the transformation rule among precipitation, streamflow, and water storage for the Tibetan Plateau on a seasonal scale. We propose a precipitation memory curve to address seasonal catchment memory. The precipitation memory curve allows us to describe how precipitation is memorized by catchments and quantify the impact of precipitation on TWS change (TWSC) for different river basins and different months. To consider both the process by which catchments memorize precipitation and the intra-annual precipitation distribution, we introduce two metrics, the influence time and the domination time, to describe the duration of seasonal catchment memory.

## Results

### River basins and their classification in the Tibetan Plateau

**Fig. 1:**
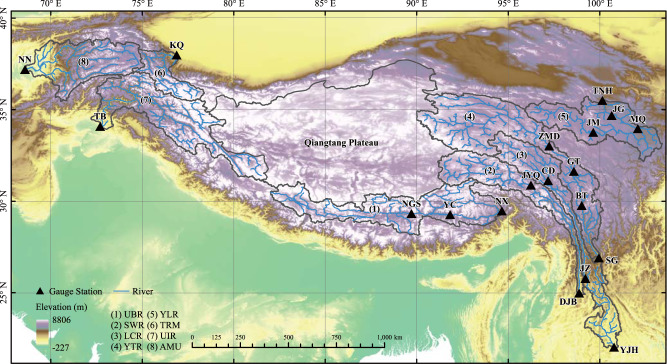
Major river basins and gauge stations in the Tibetan Plateau. The precipitation-dominated basins are the upper Brahmaputra River (UBR) basin, the Salween River (SWR) basin, the Lancang River (LCR) basin, the upper Yangtze River (YTR) basin, and the upper Yellow River (YLR) basin. The the non-precipitation-dominated basins are the upper Tarim River (TRM) basin, the upper Indus River (UIR) basin, and the upper Amu Darya (AMU) basin.

We analyzed eight upstream rivers in the Tibetan Plateau (Fig. 1), namely the upper Brahmaputra River (UBR), the Salween River (SWR), the Lancang River (LCR), the upper Yangtze River (YTR), the upper Yellow River (YLR), the upper Tarim River (TRM), the upper Indus River (UIR), and the upper Amu Darya (AMU). Among these rivers, the UIR basin has the largest percentage of glacier area (14.91%), while the YLR basin is the least affected by glaciers. Supplementary Table 1 shows the basic information of the gauge stations used for hydrological model calibration and validation in this study. The basins can be categorized into two groups. The first group is the precipitation-dominated area, including UBR, SWR, LCR, YTR and YLR, which are mainly influenced by the southwest monsoon^26^. According to monsoon variations, we divided the climate into four seasons: pre-monsoon season (April to May), monsoon season (July to September), post-monsoon season (October to November), and winter season (December to March)^27,28^. The second group is the non-precipitation-dominated area or the westerlies-dominated area, including TRM, UIR, and AMU, where the water supply depends more on glacier and snow melt than on precipitation-generated runoff^29^.

### Hydrological model calibration and validation in high-elevation rivers

We utilized a modified version of the Variable Infiltration Capacity (VIC) Macroscale Hydrological Model^30,31^, a distributed hydrological model, to simulate runoff and evapotranspiration (ET) in eight high mountain river basins. The configuration and meteorological forcing data of the VIC model are described in the Methods section. In this study, we compared the model-simulated streamflow and ET with the observed streamflow and model-based terrestrial ET from National Tibetan Plateau Data Center (see “Methods”). It should be noted that the calibration and validation periods are different among stations due to the limitation of observed streamflow data (see Supplementary Table 1). We used the multi-objective optimization algorithm, *ε*-NSGA-II, to calibrate the model (see “Methods”). Table 1 shows the results of calibration and validation in the eight basins, indicating that the VIC model can simulate streamflow and ET accurately, with NSE values mostly greater than 0.8, and BIAS values less than ±20% in both calibration and validation periods.

**Table 1:** Nash–Sutcliffe efficiency (NSE) and relative bias values (BIAS) between VIC-simulated streamflow/evapotranspiration and observed streamflow/evapotranspiration for calibration and validation periods at gauge stations in eight basins

Basin	Station	Streamflow				Evapotranspiration			
		Calibration		Validation		Calibration		Validation	
		NSE	BIAS	NSE	BIAS	NSE	BIAS	NSE	BIAS
UBR	NGS	0.87	0.10	0.73	0.20	0.92	−0.06	0.90	−0.02
	YC	0.91	−0.12	0.83	−0.05	0.92	−0.12	0.91	−0.13
	NX*	0.92	−0.08	0.88	−0.01	0.87	−0.18	0.83	−0.21
SWR	JYQ	0.92	−0.03	0.87	0.10	0.81	−0.15	0.78	−0.21
	DJB	0.92	0.04	0.93	0.02	0.81	−0.20	0.76	−0.24
LCR	CD	0.86	−0.18	0.78	−0.20	0.75	−0.26	0.79	−0.25
	JZ	0.77	−0.08	0.71	−0.15	0.78	−0.25	0.81	−0.22
	YJH*	0.90	−0.02	0.85	0.08	0.88	−0.15	0.87	−0.15
YTR	ZMD	0.81	0.15	0.78	0.18	0.89	−0.07	0.87	−0.11
	GT	0.84	0.08	0.82	0.08	0.89	−0.10	0.86	−0.13
	BT	0.87	−0.09	0.89	−0.08	0.86	−0.17	0.83	−0.19
	SG*	0.84	−0.16	0.86	−0.17	0.82	−0.21	0.77	−0.24
YLR	JM	0.70	0.25	0.54	0.39	0.72	0.16	0.76	0.08
	MQ	0.82	0.13	0.81	0.12	0.89	0.04	0.87	0.00
	JG	0.83	0.16	0.82	0.15	0.91	−0.01	0.89	−0.04
	TNH*	0.74	0.06	0.82	0.21	0.92	−0.05	0.90	−0.08
TRM	KQ*	0.90	−0.04	0.85	0.12	0.77	−0.08	0.68	−0.23
UIR	TB*	0.81	−0.05	0.75	0.13	0.60	−0.26	0.64	−0.25
AMU	NN*	0.64	−0.22	0.54	0.05	0.40	−0.39	0.46	−0.38

*UBR* the upper Brahmaputra River basin, *SWR* the Salween River basin, *LCR* the Lancang River basin, *YTR* the upper Yangtze River basin, *YLR* the upper Yellow River basin, *TRM* the upper Tarim River basin, *UIR* the upper Indus River basin, *AMU* the upper Amu Darya basin.

Note: Starred stations are the outlet stations of the basins.

**Fig. 2:**
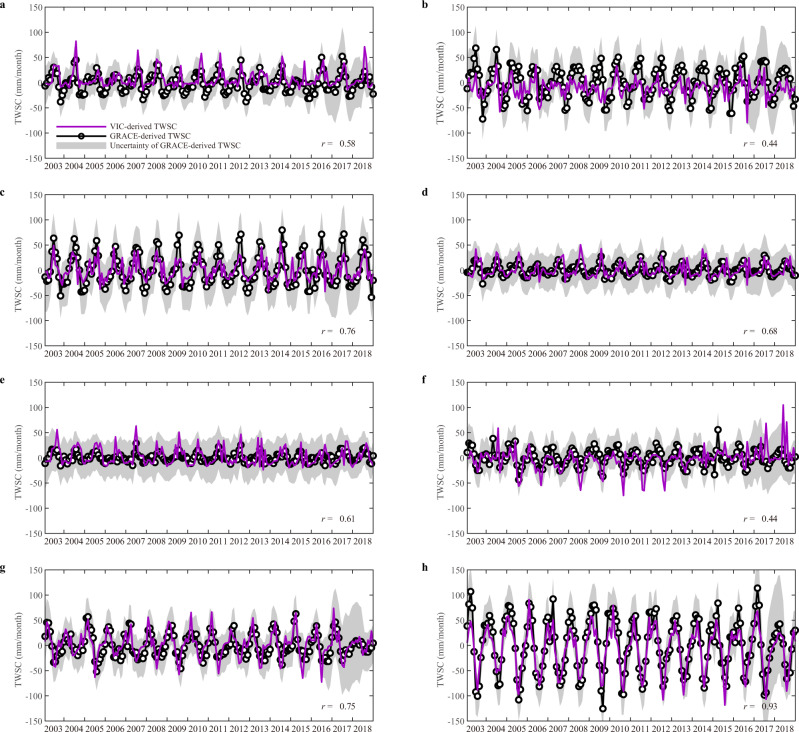
Comparison of GRACE terrestrial water storage change (TWSC) with TWSC estimated from the Variable Infiltration Capacity (VIC) model. GRACE-derived TWSC and VIC-derived TWSC for the period 2003–2018 in **a** the upper Brahmaputra River basin, **b** the Salween River basin, **c** the Lancang River basin, **d** the upper Yangtze River basin, **e** the upper Yellow River basin, **f** the upper Tarim River basin, **g** the upper Indus River basin, and **h** the upper Amu Darya basin.

We also compared the VIC-derived TWSC of the eight basins with the GRACE-based TWSC estimates from 2003 to 2018. We used the ensemble mean of TWSA from two different GRACE/GRACE-FO mass concentration (mascon) solutions, namely the Center for Space Research mascon products (CSR RL06) and Jet Propulsion Laboratory mascon solutions (JPL RL06). The data gap between GRACE and GRACE-FO in 2017 was filled with reconstructed terrestrial water storage data (see Data and data processing). The VIC-derived TWSC estimates are generally consistent with GRACE-derived TWSC estimates, with the AMU basin showing the highest value of *r* at 0.93, and the SWR basin showing the lowest value of *r* at 0.44. Figure 2 indicates that the VIC-derived TWSC values in the eight study basins are well enveloped by the uncertainty intervals of GRACE-derived TWSC, demonstrating a reasonable reproduction of TWSC in these high-elevation rivers.

### Relationship among basin water storage, streamflow, and precipitation

**Fig. 3:**
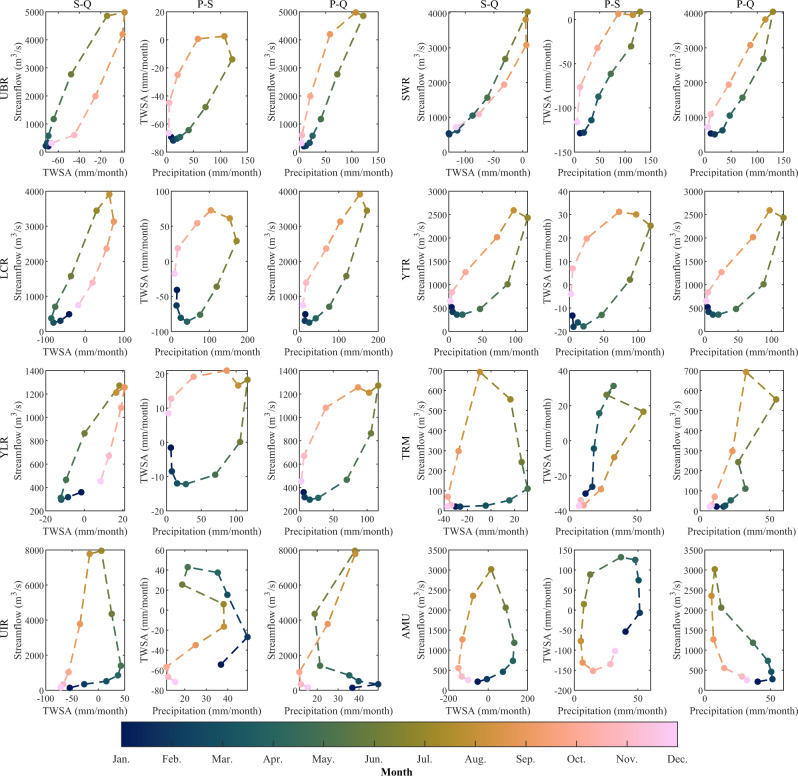
Mean annual hysteresis plots between terrestrial water storage anomalies (TWSA), streamflow, and precipitation. The TWSA-streamflow (S-Q), precipitation-TWSA (P-S), and precipitation-streamflow (P-Q) hysteresis loops are plotted with monthly mean data from 2003 to 2018 in the upper Brahmaputra River (UBR) basin, the Salween River (SWR) basin, the Lancang River (LCR) basin, the upper Yangtze River (YTR) basin, and the upper Yellow River (YLR) basin. The non-precipitation-dominated basins are the upper Tarim River (TRM) basin, the upper Indus River (UIR) basin, and the upper Amu Darya (AMU) basin.

The VIC-simulated streamflow, GRACE-derived TWSA, and precipitation from 2003 to 2018 were employed to investigate the seasonal catchment memory (Fig. 3). In the precipitation-dominated basins, our analysis revealed a positive correlation between streamflow and precipitation, and an increase in TWSA accompanied an increase in precipitation. The annual TWSA-streamflow (S-Q) clockwise hysteresis loops, the annual precipitation-TWSA (P-S) anticlockwise hysteresis loops, and the annual precipitation-streamflow (P-Q) anticlockwise hysteresis loops were observed in the five precipitation-dominated basins (Fig. 3). These three loop types illustrate the transformation rule among precipitation, streamflow, and stored water.

Two possible explanations have been identified for the P-Q anticlockwise hysteresis loops. One possible explanation is attributed to the difference in evapotranspiration (ET) between MAM and SON. However, the relative bias of ET between MAM and SON for the UBR, SWR, LCR, YTR, and YLR basins are merely 2.42%, −1.04%, −11.97%, 0.78%, and 4.60%, respectively, which suggests that ET may play a minor role in this hysteresis phenomenon.

The other explanation hypothesizes that the precipitation in the pre-monsoon and monsoon seasons is retained within the basin, and subsequently, the stored water is gradually released into the streamflow. The P-S anticlockwise hysteresis loops confirm the temporary storage of precipitation within the catchment. The observed change in TWSA provides insights into the destination/source of the missing/excess streamflow associated with the same amount of precipitation in the pre-monsoon and monsoon seasons. This finding is consistent with ref. ^15^, where TWS is used as a catchment memory module. The S-Q clockwise hysteresis loops indicate that the processing of water storage and release within the catchment can affect the rate of streamflow increase and decrease, although precipitation remains the primary driver of streamflow variability.

Moreover, in the same basin, the P-Q loop exhibits a narrower range of variability than the P-S loop, which suggests that streamflow responds to changes in precipitation more rapidly than TWSA. This implies that a larger proportion of precipitation generates runoff within a month, while only a small fraction of precipitation is memorized through water storage. The shape of hysteresis loops for a basin can represent the feature of the precipitation-runoff relationship. From the hysteresis loops, we found that the catchment can store a portion of precipitation and then release the stored water to influence the streamflow on a monthly scale. This confirms the existence of seasonal catchment memory in precipitation-dominated basins.

**Fig. 4:**
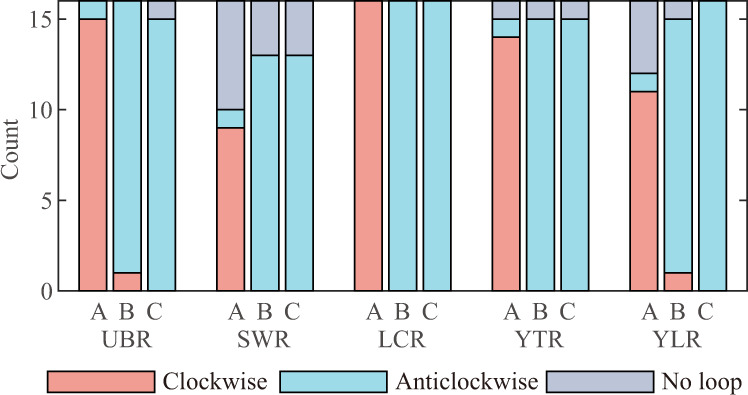
Direction statistics of the annual hysteresis loops from 2003 to 2018 for the five precipitation-dominated basins. A, B, and C represent the TWSA-streamflow (S-Q), precipitation-TWSA (P-S), and precipitation-streamflow (P-Q) hysteresis loop, respectively. UBR upper Brahmaputra River basin, SWR Salween River basin, LCR Lancang River basin, YTR upper Yangtze River basin, YLR upper Yellow River basin.

There are still certain exceptions in the annual hysteresis loops in some years, i.e., no clear hysteresis loop, the S-Q anticlockwise hysteresis loop, the P-S clockwise hysteresis loops, and the P-Q clockwise hysteresis loop. Supplementary Figs. 2–6 display the annual hysteresis loops from 2003 to 2018 for the five precipitation-dominated basins. S-Q clockwise hysteresis loops, P-S anticlockwise hysteresis loops and P-Q anticlockwise hysteresis loops are observed in the majority of cases (Fig. 4). It is worth noting that the S-Q loops are most susceptible to exceptions, as both TWSA and streamflow are dominated by precipitation. In the UBR basin, all three loops in 2015 are abnormal. Although the rotation directions of three loops in 2009 can be identified, they resemble more of a curve than a loop. The precipitation anomaly is the primary contributor to the loop anomaly in 2009 and 2015. Specifically, the precipitation in 2009 is the lowest in 16 years, representing a 26% reduction compared to the 16-year average (Supplementary Fig. 7). In 2015, the precipitation is approximately 16% less than the 16-year average, with precipitation in July being 58% less than the 16-year average precipitation in July. In the SWR basin, the hysteresis loops are relatively narrow, and their direction is most susceptible to changes among the five basins. In 2006, the S-Q anticlockwise hysteresis loop is observed in the SWR basin, with precipitation during monsoon season being ~21% less than the 16-year average precipitation. In some years, such as 2005, 2009 and 2015, a dry September (19%, 39%, and 29% less than the average, respectively) may cause the loop line to cross. The intra-annual changes in precipitation distribution may also result in the disappearance of the hysteresis loops, as demonstrated by the case of 2010 and 2011. In the YTR basin, the direction of S-Q hysteresis loop of 2015 is anticlockwise, which can also be attributed to the precipitation anomaly. The precipitation is ~22% less than the average during monsoon season, with precipitation in July being 50% less than the average in 2015. In the YLR basin, TWSA and streamflow show no clear hysteresis loop and an anticlockwise hysteresis loop in 2010 and 2015, respectively. TWSA in 2010 and 2015 decreases rapidly from July to August, with the precipitation being ~40% less than the 16-year average precipitation in July and August. It is observed that TWSA in 2006 has a decrease from 10.1 mm in January to −9.9 mm in December, which continues into May of 2007 (Supplementary Fig. 8). This phenomenon may be associated with a 45% reduction of precipitation but a 77% increasing of ET in May of 2006 (Supplementary Fig. 7).

Generally, the relationship between TWSA and streamflow in the precipitation-dominated basins follows a clockwise hysteresis loop. In the S-Q anticlockwise hysteresis loops, we observed that TWSA in July was greater than TWSA in August, which means that TWSC in July needs to be less than 0. According to Eq. (4), a decreasing precipitation, an increasing streamflow, or an increasing ET can result in a negative TWSC. Our results demonstrate that ET remained relatively constant, particularly during monsoon season, whereas streamflow varied in the same trend as precipitation (Supplementary Fig. 7). Furthermore, we found that a decrease in precipitation during July is frequently associated with a S-Q clockwise hysteresis loop. Therefore, we supposed that a reduction in precipitation, particularly in July, can lead to S-Q anticlockwise hysteresis loops, while the rotation directions of P-Q and P-S hysteresis loops are relatively stable.

In the non-precipitation-dominated basins, the relationships among basin water storage, streamflow, and precipitation differ from those of the precipitation-dominated basins (Fig. 3). In the UIR and AMU basins, increasing precipitation does not correspond to increasing streamflow. We observe that precipitation in the UIR and AMU basins is concentrated from January to April. Despite large precipitation, streamflow does not increase, but TWSA rises sharply. This suggests that a substantial proportion of precipitation is stored in the basins as ice and snow. A similar pattern is found in the TRM basin, where TWSA shows a clear increase from January to April, but streamflow does not. Unlike the other two basins, the continued increase in precipitation in the TRM basin has resulted in a clear increase in both streamflow and TWSA. However, the TRM basin still differs from the precipitation-dominated basins, as the direction of the P-S hysteresis loop is clockwise. This implies that the amount of stored water from the large precipitation in July is less than the amount of released water (i.e., melting water). The anticlockwise S-Q loops in all three non-precipitation-dominated basins suggest that a large portion of streamflow originates from TWSA during June to September. Nonetheless, the observed positive correlation between streamflow and precipitation, coupled with the presence of an anticlockwise P-Q hysteresis loop in the TRM basin, suggests the potential existence of a precipitation memory effect during the warm season (from June to September). We also verified the relationships among TWSA, streamflow, and temperature in the three basins (see Supplementary Fig. 1). Our analysis shows a high correlation between temperature and streamflow with almost no hysteresis. The temperature-TWSA (T-S) loops show that substantial quantities of water are stored in the basins as ice and snow from January to April (when the temperature is below 0 °C), and then released from June to September (when the temperature is above 0 °C). Generally, in the non-precipitation-dominated basins, changes in TWSA and streamflow are mainly influenced by temperature. In the TRM basin, the seasonal catchment memory of precipitation may exist in warm seasons, whereas in the UIR and AMU basins, meltwater controlled by temperature substantially weakens this seasonal catchment memory of precipitation.

### Curve for a catchment memory process

**Fig. 5:**
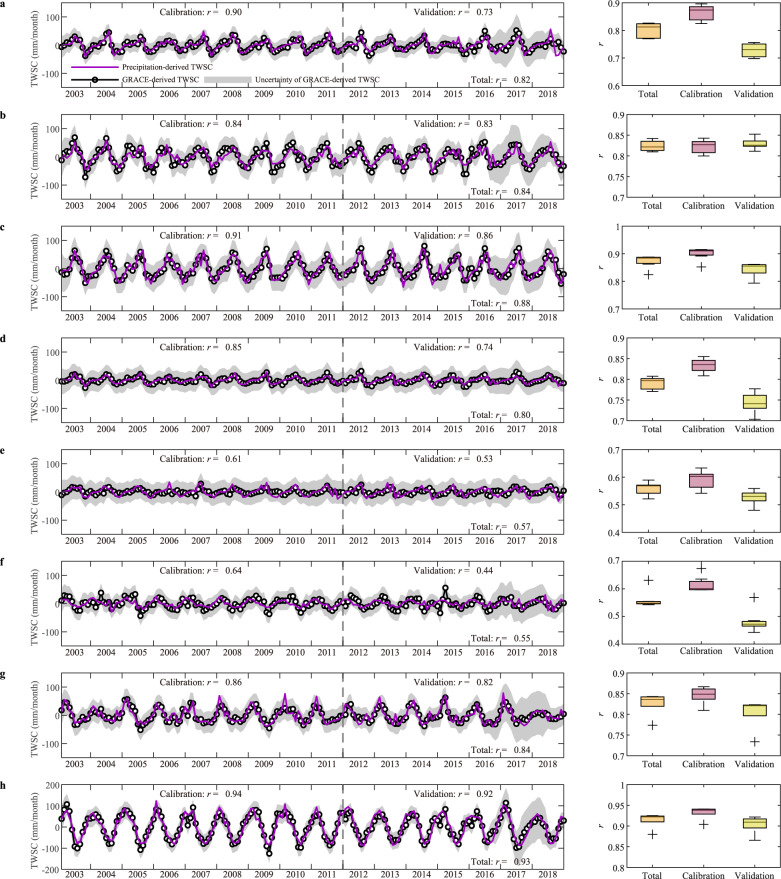
Comparison of GRACE terrestrial water storage change (TWSC) with TWSC estimated from the precipitation-to-TWSC model. The left panel shows the GRACE-derived TWSC and precipitation-derived TWSC during the period 2003–2018 and the right panel shows the performance of TWSC estimated with different precipitation products in **a** the upper Brahmaputra River basin, **b** the Salween River basin, **c** the Lancang River basin, **d** the upper Yangtze River basin, **e** the upper Yellow River basin, **f** the upper Tarim River basin, **g** the upper Indus River basin, and **h** the upper Amu Darya basin. The boxplots’ lower whisker, lower box edge, middle line, upper box edge, and upper whisker represent 10th, 25th, 50th, 75th, and 90th percentiles, respectively. The plus sign represents the outlier.

Here, we utilized the newly proposed precipitation-to-TWSC model (see “Methods”) to obtain the precipitation memory curve, which describes the catchment memory process. In the non-precipitation-dominated basins, we employed a revised model with a temperature-index function (see “Methods”) to account for the critical role of temperature. As shown in Fig. 5, the precipitation-to-TWSC model accurately reproduces the basin water storage variability with precipitation data. The analysis of the model’s uncertainty can be found in the Methods section. Comparing the VIC-derived TWSC in Fig. 2, the precipitation-simulated TWSC demonstrates clear improvement in most basins, with the correlation coefficients increasing from >0.5 to >0.8. This result suggests that the precipitation memory curve obtained from the model for both precipitation-dominated and no-precipitation-dominated basins can be deemed reliable.

**Fig. 6:**
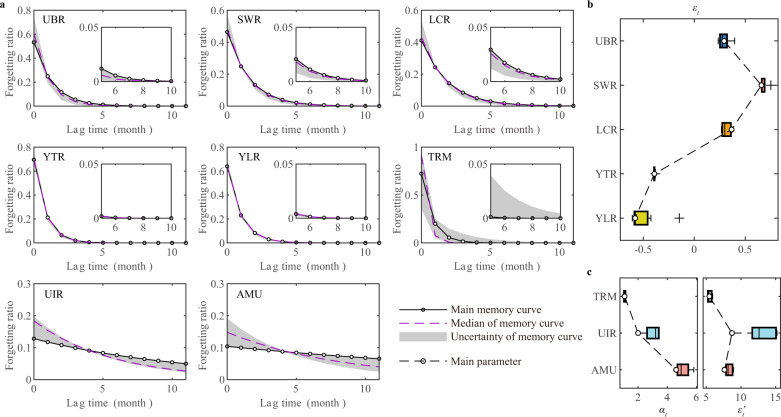
Distribution of parameters for the precipitation-to-TWSC (terrestrial water storage change) model. **a** precipitation memory curve with a lag time from 0 to 11 months. The uncertainty boundaries of memory curves are the maximum and minimum values in the set of curves with different precipitation products, excluding outliers of the shape parameter *b*_*t*_. **b** values of the paramter *ε*_*t*_, which represents additional water fluxes participating in the water balance except precipitation and evapotranspiration (ET). **c** values of the parameter *α*_*t*_ and *ε’*_*t*_, which are used in the temperature-index function to update *ε*_*t*_. UBR upper Brahmaputra River basin, SWR Salween River basin, LCR Lancang River basin, YTR upper Yangtze River basin, YLR upper Yellow River basin, TRM upper Tarim River basin, UIR upper Indus River basin, AMU upper Amu Darya basin. The main parameters are the results from the main precipitation products. For UBR, SWR, LCR, YTR, YLR and TRM, the main precipitation product is the China Meteorological Forcing Dataset (CMFD). The temperature data for TRM are also from CMFD. For UIR and AMU, the main precipitation product is the Integrated Multi-satellitE Retrievals for GPM Final Run Version 6 dataset (IMERG) and the temperature data are from the Global Land Data Assimilation System dataset (GLDAS). The boxplots’ lower whisker, lower box edge, middle line, upper box edge, and upper whisker represent 10th, 25th, 50th, 75th, and 90th percentiles, respectively. The plus sign represents the outlier.

Based on the precipitation memory curves, the five precipitation-dominated basins are grouped into two categories. The first category includes the UBR, SWR, and LCR basins, where the forgetting ratio of precipitation at 0-month lag is relatively low and the catchment memory lasts for a long time. The YTR and YLR basins belong to the second category, with a short precipitation memory duration of less than 4 months. Although the two categories of basins differ in their precipitation memory durations, less than 10% of the precipitation can be memorized in the catchment after 4 months for all five basins. Interestingly, basins in the first category all have a positive additional flux, ε_*t*_, while those in the second category all have negative values (Fig. 6b). The Mann–Kendall method^32^ was used to investigate the cause of this phenomenon by examining the trends of GRACE-derived TWSA, VIC-simulated streamflow, surface precipitation, and VIC-simulated areal evaporation (Supplementary Table 2). The first category corresponds to the decreasing TWSA, while the second category corresponds to increasing TWSA. The detected mass loss signals in the UBR, SWR, and LCR basins are in agreement with the findings of previous work^33,34^. The trend test results show that there is no significant decreasing or increasing trend in the corresponding streamflow, precipitation and ET in the UBR, SWR, and LCR basins. However, the joint effects of insignificant decreasing precipitation, insignificant increasing ET and rising temperature are supposed to be the cause of decreasing TWSA^35,36^. Jing et al. noted that the successfully preserved groundwater in the Chinese Ecological Protection and Construction Project (CEPCP) causes increasing TWSA of the YTR and YLR basins^33^. In the study of ref. ^37^, a different interpretation is proposed that High Mountain Asia blocks the propagation of westerlies-carried deficit in precipitation minus evaporation from the southeast North Atlantic into the central Tibetan Plateau, causing a monthly TWS increase.

In the non-precipitation-dominated basins, we identified two distinct shapes. Specifically, the TRM basin is characterized by a steep curve, while the UIR and AMU basins have flat curves. The precipitation memory curve of the TRM basin resembles those of the YTR and YLR basins, with a shorter precipitation memory duration of ~3 months. During the cold season (January to April, when the temperature is less than 0°C), ε_*t*_ in the TRM basin is negative and has a large absolute value, indicating that almost all precipitation in the TRM basin is stored in TWS. Consequently, the precipitation memory curve of the TRM basin only describes the catchment memory process from June to September, when the temperature is above 0°C. Meanwhile, the flat curves of the UIR and AMU basins suggest that seasonal catchment memory is weak due to the dominance of meltwater in these two basins. These curves, however, clearly demonstrate the existence of long-term catchment memory in the basins. This finding is consistent with the previous analysis of the hysteresis loop.

### Seasonal catchment memory duration

**Fig. 7:**
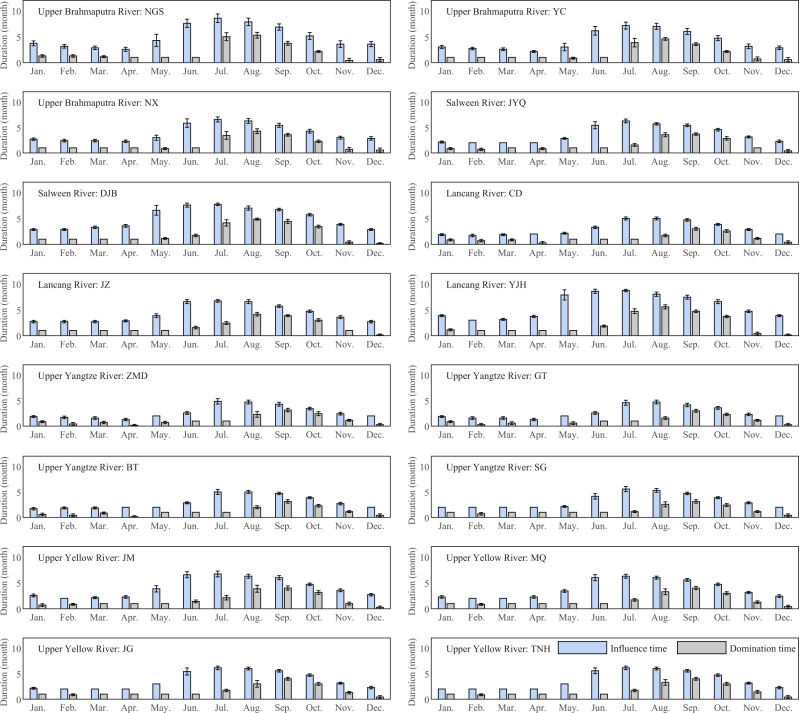
Seasonal catchment memory duration in different months in the precipitation-dominated basins. The influence time and domination time in the 16 sub-basins in the Tibetan Plateau are the mean value of results from seven precipitation products. The error bar is the standard deviation of the influence/domination time from seven precipitation products, representing the uncertainty of the influence/domination time.

In precipitation-dominated basins, the catchment memory process for monthly precipitation can be well described by the precipitation memory curve. However, the amount of precipitation in a given month determines the volume of water released in the following months, despite the basin sharing the same memory curve in different months. Hence, we introduced the influence time and the domination time, which consider both the amount of precipitation and its memory curve, to quantify catchment memory duration (see “Methods”). We calculated the influence time and the domination time for all the sub-basins above the outlet stations (see Fig. 7), and the performance of the precipitation-to-TWSC model for all these sub-basins can be seen in Supplementary Fig. 9. It demonstrates that the influence time increases from May in the pre-monsoon season, and decreases from September or October in the post-monsoon season. Conversely, the domination time increases from June, which is slightly delayed compared to the influence time. The influence time during monsoon season is ~6 months, whereas the domination time is around 4 months. During the winter season, the influence time and the domination time are about 3 months and 1 month, respectively. Although the precipitation in the winter seasons can be stored as a form of ice and snow, allowing the stored water to be released slowly, the amount of precipitation in winter is significantly smaller than that in the monsoon season. Therefore, the precipitation in the monsoon season has a larger contribution and a longer influence time to TWSC.

The distributions of the influence time and the domination time vary among basins due to differences in precipitation pattern and memory curves. Notably, the influence time and the domination time of May in DJB and YJH are larger than those of May in the other sub-basins. These two stations are located further south than the other stations and are more strongly influenced by the monsoon. However, in general, our results are consistent with the features of longer influence and domination times during monsoon season and shorter times during winter season at the same station.

The influence time and the domination time of the downstream are found to be larger than those of the upstream in the SWR basin and the LCR basin. The extension of influence time in the winter season is likely attributed to changes in the amount and distribution of precipitation from upstream to downstream, where the proportion of monsoon season’s precipitation to annual precipitation from upstream to downstream decreases from 73 to 64% in the SWR basin, and from 72 to 67% in the LCR basin. In addition, the landcover changes from grassland upstream to wooded grassland downstream, which improves the water storage capacity of downstream. Consequently, the precipitation memory curve changes and the memory duration increases. However, in the UBR, YTR and YLR basins, no clear difference in the influence time and the domination time between the sub-basins from upstream to downstream is observed because the hydro-physical conditions (such as landcover, soil type and climate condition) from upstream to downstream in the UBR, YTR and YLR basins, do not have changes as large as those of the SWR or the LCR basins (Supplementary Tables 3 and 4).

As the flat curves in the UIR and AMU basins imply long-term catchment memory which is not the main concern of our study, the influence time and the domination time for these two basins are not discussed here. In the TRM basin, it is observed that the influence time and the domination time are similar to those of winter seasons in the precipitation-dominated basins, with around 3 months and 1 month, respectively (Supplementary Fig. 10). This finding is also consistent with the steep memory curve in the TRM basin.

## Discussion

This study aimed to investigate the seasonal catchment memory in the Tibetan Plateau using terrestrial water storage data. We classified the studied basins into two categories, precipitation-dominated basins and non-precipitation-dominated basins. The obtained results have several substantial implications.

First, in the precipitation-dominated basins, the direction of the Q-S hysteresis loop is more vulnerable to disruption due to precipitation reduction during the monsoon season compared to P-Q and P-S loops. Furthermore, the shapes of hysteresis loops are determined by the basin’s runoff generation features, whereby precipitation has a dominant effect on the changes of both TWSA and streamflow. Although hysteresis loop anomalies may be caused by climate anomalies (such as reduced precipitation) or human activities (which leads to sudden changes in runoff and water storage), the direction of hysteresis loops remains unchanged in the long term due to the basin’s water storage capacity.

Second, non-precipitation-dominated basins exhibit a different relationship among precipitation, streamflow, and TWSA. Dominated by westerlies, precipitation is concentrated from January to April in these basins, corresponding to the end of winter season and the beginning of pre-monsoon season for precipitation-dominated basins. However, this precipitation does not cause an increase in streamflow but rather an increase in TWSA. This is because precipitation mainly occurs in winter in the form of snowfall, which serves as the main mass source for the glacier. A large proportion of precipitation is converted into storage in the form of snow and ice during this period. This finding agrees with the previous study indicating that the snow water equivalent dominates the seasonal fluctuations of TWS^38^. Moreover, a strong correlation exists between temperature and streamflow/TWSA within these basins. This can be explained by the fact that the UIR basin is more vulnerable to glacier loss and the AMU basin is highly dependent on snow melt^25^. In the TRM basin, meltwater and precipitation account for about 65% and 35% of streamflow, respectively^39^. As revealed by the five hysteresis loops (Q-S, T-Q, T-S, P-Q, and P-S), the TRM basin is dominated by precipitation and meltwater, while the UIR and AMU basins are mainly dominated by meltwater.

Third, the hysteresis loops in the precipitation-dominated basins indicate that the catchment can memorize antecedent precipitation and respond to it in the following period. Based on this, we proposed a precipitation-to-TWSC model that splits precipitation into two parts, with one converted into runoff and the other temporarily stored in the basin. We used a precipitation memory curve to describe the water-storing and releasing process. The model accurately estimated TWSC and fitted well with GRACE-derived TWSC during the periods from 2003 to 2018, with the highest correlation coefficient of 0.88 in the LCR basin, and the lowest correlation coefficient of 0.57 in the YLR basin. Considering the critical role of temperature in non-precipitation-dominated basins, we used a temperature-index function to update the precipitation-to-TWSC model. The accurate TWSC estimations with the correlation coefficient of 0.93 in the AMU basin were obtained with the revised model. The proposed precipitation-to-TWSC model can simulate more accurate TWSC results than the VIC model built in most basins, which indicates that the precipitation memory curve from the model is reliable.

Finally, we defined the influence time and the domination time to explore the seasonal catchment memory in the precipitation-dominated basins. These two metrics consider the process of precipitation memory on a seasonal scale and the intra-annual precipitation distribution. From the results of 16 sub-basins, we found that a longer influence time corresponds to a longer domination time during monsoon season, with an influence time of about 6 months and a domination time of about 4 months. In the winter season, the influence time and the domination time are 3 months and 1 month, respectively. One of potential applications of these results is to identify efficient lead times required for seasonal streamflow forecasts, where the precipitation in the winter season has little impact on the monsoon season’s streamflow and the precipitation in the monsoon season may dominate the streamflow in the winter season.

## Methods

### Hydrological modeling

We use a modified version of the distributed hydrological model, the Variable Infiltration Capacity (VIC) Macroscale Hydrological Model^30,31^ to simulate the runoff and evapotranspiration in eight high mountain river basins. The snow^40,41^ and frozen soil algorithms^42,43^ are included in the model to simulate snow and frozen soil appropriately in cold mountain regions. The VIC model of version 4.2.d is operated at a 6-hourly time step in both water and energy balance models with a spatial resolution of 0.25° × 0.25° in this study. In this version of VIC, glacier simulation is absent. Therefore, we add a glacier module based on a simple temperature-index model into the original VIC model^26,44,45^. The glacier melting water within Cell *i G*_*i*_ is calculated as follows:

1 $${G}_{i}=\bigg\{\begin{array}{cc}{f}_{i}\times ({D}_{f}\times {T}_{i}) &,\,if{T}_{i}\ge {T}_{melt}\\ 0 &,\,if\,{T}_{i} < {T}_{melt}\end{array}$$

Where *f*_*i*_ is the glacier area fraction in Cell *i*; *D*_*f*_ is the degree-day factor for glacier (mm °C^−1^ day^−1^); *T*_*i*_ and *T*_*melt*_ are the daily mean air temperature and the minimum temperature when the glacier melts, respectively.

The VIC model is calibrated with a global multi-objective optimization algorithm, the ε-Dominance Non-Dominated Sorted Genetic Algorithm II (ε-NSGA-II)^46,47^. Evaluation criteria including Nash–Sutcliffe efficiency (NSE) coefficient^48^ and relative bias (BIAS) are chosen to evaluate the simulated performance of streamflow and ET. The parameters selected for calibration in this study are the thickness of the second and third soil moisture layer (*d*_2_, *d*_3_), the fraction of maximum soil moisture where nonlinear baseflow occurs (*Ws*), the maximum velocity of baseflow (*Ds*_*max*_), the fraction of *Ds*_*max*_ where nonlinear baseflow begins (*Ds*), the variable infiltration curve parameter (*b*_*inf*_), and the degree-day factor for glacier (*D*_*f*_)^49,50^.

### Derivation of TWSC and its uncertainty

GRACE-derived TWSC is estimated by the double difference time derivative of TWSA^51,52^ as follows:

2 $$TWSC(t)=\frac{TWSA(t+1)-TWSA(t-1)}{2}$$

Where *TWSC*(*t*) is terrestrial water storage change for Time *t* across the study basin; *TWSA*(*t* + 1) and *TWSA*(*t* − 1) are terrestrial water storage anomalies for Time *t* + 1 and *t*−1, respectively.

With the uncertainty of GRACE-derived TWSA, the uncertainty of TWSC can be calculated as follows:

3 $${U}_{TWSC}(t)=\frac{{U}_{TWSA}(t+1)+2\ast {U}_{TWSA}(t)+{U}_{TWSA}(t-1)}{4}$$

Where *U*_*TWSC*_(*t*) is the uncertainty of TWSC for Time *t*; *U*_*TWSA*_(*t*) is the uncertainty of TWSA for Time *t*.

In a closed basin, VIC-derived TWSC is estimated using the water budget approach^53,54^. The mass conservation equation for estimating the change in water storage at Time *t* is:

4 $$TWSC(t)=P(t)-ET(t)-R(t)$$

Where *R* is VIC-simulated runoff, *P* is precipitation data, *ET* is VIC-simulated evapotranspiration. The correlation coefficient (*r*) is used to evaluate the performance of VIC-derived TWSC.

### Precipitation-to-TWSC conceptual model

In general, part of precipitation generates runoff directly when it falls, while the other part is stored in the form of snow, ice, groundwater, and soil water, and then slowly released as runoff. Thus, the monthly precipitation at Time *t*, *P*(*t*), can be represented as the following equation.

5 $$P(t)=\mathop{\sum }\limits_{\varDelta t=0}^{n}{P}_{t}(\varDelta t),\,\varDelta t=0,\,1,{{{{\mathrm{..}}}}}.,\,n$$

Where Δ*t* is a lag time; *P*_*t*_(Δ*t*) represents the water released from *P*(*t*) at the *t* + Δ*t* time; *n* is the longest time for the precipitation memorizing.

With Eq. (5) and the water budget equation in a closed basin, we estimate the monthly streamflow at the outlet station as follows:

6 $$R(t)=\mathop{\sum }\limits_{\varDelta t=0}^{n}{P}_{t-\varDelta t}(\varDelta t)-ET(t)+{\varepsilon }_{t}$$

Where *R*(*t*) and *ET*(*t*) are the runoff and evapotranspiration at Time *t*, respectively; ε_*t*_, represents additional water fluxes participating in the water balance except precipitation and ET, such as ancient glacier melting/replenishment, deep groundwater discharge/recharge, artificial water intake/input, etc.; the first item in the right of Eq. (6) means the total amount of water released from the precipitation at Time *t*.

With Eq. (6) replacing the item *R*(*t*) in Eq. (4), we derive TWSC as the following:

7 $$TWSC(t)=P(t)-\mathop{\sum }\limits_{\varDelta t=0}^{n}{P}_{t-\varDelta t}(\varDelta t)-{\varepsilon }_{t}$$

Here, an index is used to relate *P*(*t*) to *P*_*t*_(Δ*t*) as the following:

8 $${P}_{t}(\varDelta t)={c}_{t,\varDelta t}P(t),\,where\,\mathop{\sum }\limits_{\varDelta t=0}^{n}{c}_{\varDelta t,t}=1$$

Where *c*_*t*,Δ*t*_ is the recession index at Time *t* with a Δ*t* lag time, namely forgetting ratio. Assuming *c*_*t*,Δ*t*_ decays with time, we propose a precipitation memory function with reference to the hydrograph recession function^55^.

9 $${c}_{t,\varDelta t}=\frac{{e}^{-{b}_{t}\varDelta t}}{\mathop{\sum }\limits_{\varDelta t=0}^{n}{e}^{-{b}_{t}\varDelta t}}$$

Where *b*_*t*_ is a coefficient to decide the shape of the precipitation memory curve at Time *t*; *e* is the natural constant. In this study, the longest time for precipitation memorizing is set as 1 year, i.e., *n* = 11. Due to the limited data, when considering that *b*_*t*_ and ε_*t*_ are time-varying, it is hardly to avoid a large parameter uncertainty. Therefore, in this study, the parameters, *b*_*t*_ and *ε*_*t*_, are considered as time-invariant parameters in the non-precipitation-dominated river basins.

In westerlies-dominated arid river basins, water supply is more dependent on glacier and snow melts than precipitation-generated runoff. Referring to the degree-day factor model, we introduce a temperature-index function to update the factor *ε*_*t*_ in Eq. (6) and Eq. (7) as the following:

10 $${\varepsilon }_{t}={\alpha }_{t}{T}_{t}+{\varepsilon ^{\prime} }_{\!\!t}$$

Where *α*_*t*_ is a degree factor, *T*_*t*_ is monthly mean air temperature, and *ε’*_*t*_ represents fluxes participating in the water balance excluding the glacier and snow melts. When *T*_*t*_ is below 0, the precipitation is stored as snow and ice; otherwise, the streamflow is supplemented by snow and ice melt.

NSGA-II^56,57^ is used to calibrate the precipitation-to-TWSC model parameters, in which the calibration period is from 2003 to 2011 and the validation period is from 2012 to 2018. We employ six additional precipitation datasets (see Data and data processing) to evaluate the model uncertainty introduced by precipitation uncertainty (the right panel of Fig. 5). In the eight studied basins, the performance of the model using different precipitation datasets as input is very close, where the uncertainties are all less than 0.1. This indicates that the uncertainty of precipitation has little impact on the performance of the model. Except the TRM basin, we also find that the uncertainty of precipitation has little impact on the model parameters of other basins (Fig. 6a).

### Metrics for seasonal catchment memory

In this study, the influence time and the domination time are proposed to describe the seasonal catchment memory duration. According to the total amount of water released from the precipitation at Time *t* in Eq. (6), the contribution rate of precipitation at Time *t*-Δ*t* to the precipitation-releasing water amount at Time *t* can be calculated as the following:

11 $$C{R}_{t,\varDelta t}=\frac{{c}_{t-\varDelta t,\varDelta t}P(t-\varDelta t)}{\mathop{\sum }\limits_{\varDelta t=0}^{n}{c}_{t-\varDelta t,\varDelta t}P(t-\varDelta t)}$$

The lag time Δ*t* satisfying Eq. (12) is defined as the influence time of precipitation at Time *t*, which represents the longest time that the precipitation at Time *t* can influence TWSC. The lag time Δ*t* satisfying Eq. (13) is defined as the domination time of the precipitation at Time *t*, which indicates the longest time that the precipitation at Time *t* plays a leading role in TWSC.

12 $$C{R}_{t,\varDelta t}\,\ge \,1\%,\,C{R}_{t,\varDelta t+1}\, < \,1\%$$

13 $$C{R}_{t,\varDelta t}\,\ge \,10\%,\,C{R}_{t,\varDelta t+1}\, < \,10\%$$

### Data and data processing

The China Meteorological Forcing Dataset (CMFD) is used as the forcing data of the VIC model^58,59^. CMFD is a high spatial-temporal resolution gridded near-surface meteorological dataset, including 2-m air temperature, surface pressure, specific humidity, 10-meter wind speed, downward shortwave radiation, downward longwave radiation and precipitation rate, with the resolution of 0.1° × 0.1°. We resample the dataset into 0.25° × 0.25° with cubic convolution interpolation. These meteorological data are from 1999 to 2018. However, the CMFD data do not cover two basins, i.e., UIR and AMU. As a result, the precipitation data for these two basins are from the Integrated Multi-satellitE Retrievals for GPM Final Run Version 6 (IMERG) dataset, and all the other meteorological data are from the Global Land Data Assimilation System (GLDAS) dataset. Supplementary Fig. 11 shows the consistency analysis results (correlations) between precipitation data of CMFD and IMERG, and temperature data of CMFD and GLDAS. Both precipitation data and temperature data from different sources show strong correlations. In addition, the model parameters calibration can reduce systematic bias in meteorological input and improve the hydrological model’s tolerance to meteorological errors from different sources^60^. The streamflow data in China are from the gauged hydrological stations. The streamflow data of UIR and AMU are from Pakistan Water and Power Development Authority (WAPDA) and Third Pole Environment Data Center (TPDC), respectively. The calibration and validation periods for each river basin are shown in Supplementary Table 1.

The precipitation datasets used to estimate TWSC in this study is CMFD, the China Gauge-based Daily Precipitation Analysis (CGDPA)^61,62^, the Tropical Rainfall Measuring Mission 3b42v7 (TRMM), IMERG, the ECMWF Reanalysis v5 monthly averaged data (ERA5), the Climate Prediction Center Morphing Technique Climate Data Record (CMORPH) and the Precipitation Estimation from Remotely Sensed Information using Artificial Neural Networks—Climate Data Record (PERSIANN). The basic information of these datasets is shown in Supplementary Table 5. In the UIR and AMU basins, the input temperature data for the precipitation-to-TWSC model are from GLDAS dataset.

The digital elevation model data with 90 m resolution is from National Aeronautics and Space Administration (NASA) Shuttle Radar Topographic Mission (SRTM). Harmonized World Soil Database (HWSD)^63^ in a resolution of 1 km is adopted as the soil dataset in the hydrological model. Landcover datasets used in our work are the WestDC landcover dataset version 2.0^64^ and Global Land Cover 2000 Project (GLC2000) dataset in a resolution of 1 km in the year of 2000. Glacier data are from the Second Glacier Inventory of China^65^ and WestDC Global Glacier dataset.

The monthly 0.01° terrestrial evapotranspiration dataset over the Tibetan Plateau from 2000 to 2018 (TED-TP) is used in this study^66^. The multiyear (2000–2018) monthly ET in the Tibetan Plateau are estimated using the MOD16-STM model supported by NDVI data, CMFD meteorological data, ERA5 land surface temperature data, GLASS albedo data, GLEAM topsoil moisture data, HWSD data and verified by 9 flux towers in the Tibetan Plateau. Since the ET data in the lower LCR is absent, the harmonized global land ET product with the reliability ensemble averaging method (REA-ET)^67^ is employed in the LCR basin. The dataset is combined based on three model-based products, i.e., ERA5, GLDAS and MERRA-2, with a spatial resolution of 0.25° × 0.25°. The global ET dataset based on ETMonitor model (ETM)^68^ with 1 km resolution is employed in the UIR and AMU basins, which has been validated by ground measurements from 251 flux towers across various ecosystems and climate zones globally.

The GRACE data used in this study are Center for Space Research (CSR RL06) mascon products and Jet Propulsion Laboratory (JPL RL06) mascon solutions. There is a slight difference between TWSA products derived from CSR and JPL for the various choices of parameters and solution methods^54^. A rough value of 20 mm recommended by the CSR website (http://www2.csr.utexas.edu/grace) is used as the uncertainty of CSR data and the uncertainty of JPL data is estimated with the methods described in ref. ^69^. The missing data due to battery management during the study periods are filled by linearly interpolating the values of adjacent months. For basins in China, the data gap between GRACE and GRACE-FO in 2017 are filled with the dataset of reconstructed terrestrial water storage in China based on precipitation^70^, which uses CGDPA and CN05.1 temperature data to reconstruct the CSR and JPL Mascon solutions. The bias of most regions in China is within 5 cm. For UIR and AMU, we use the global reconstructed JPL data^71^ and the global reconstructed CSR data^72^ to fill the data gap.

## Supplementary information

Supplementary Information

## Data Availability

The data produced by this study are available at 10.6084/m9.figshare.22769675. GRACE and GRACE-FO data are provided by the NASA MEaSUREs Program, among which JPL mascon data can be accessed at https://grace.jpl.nasa.gov/ and the CSR mascon data can be accessed at http://www2.csr.utexas.edu/grace. The dataset of reconstructed terrestrial water storage in China based on precipitation can be accessed at 10.11888/Hydro.tpdc.270990. The global reconstructed JPL mascon data can be accessed at 10.6084/m9.figshare.7670849.v3. The global reconstructed CSR mascon data can be accessed at 10.5061/dryad.z612jm6bt. SRTM DEM data are downloaded from https://earthexplorer.usgs.gov/. Soil data are downloaded from https://www.fao.org/soils-portal/soil-survey/soil-maps-and-databases/harmonized-world-soil-database-v12/en/. GLC2000 data are downloaded from https://forobs.jrc.ec.europa.eu/products/glc2000/glc2000.php. WestDC landcover data are downloaded from https://cstr.escience.org.cn/CSTR:11738.11.ncdc.Westdc.2020.678. The Second Glacier Inventory of China can be accessed at 10.3972/glacier.001.2013.db. The WestDC Global Glacier dataset can be accessed at https://cstr.escience.org.cn/CSTR:11738.11.ncdc.Westdc.2020.764. CMFD data are downloaded from 10.11888/AtmosphericPhysics.tpe.249369.file. TRMM data are downloaded from https://disc.gsfc.nasa.gov/datasets/TRMM_3B42_Daily_7/ summary. IMERG are downloaded from https://gpm.nasa.gov/data/imerg. ERA5 data are downloaded from https://cds.climate.copernicus.eu/cdsapp#!/dataset/reanalysis-era5-land-monthly-means. CMORPH data can are downloaded from https://www.ncei.noaa.gov/products/climate-data-records/precipitation-cmorph. PERSIANN data are downloaded from https://www.ncei.noaa.gov/products/climate-data-records/precipitation-cmorph. CGDPA data can be accessed at http://data.cma.cn. GLDAS data are downloaded from https://disc.gsfc.nasa.gov/datasets/GLDAS_NOAH025_3H_2.1/summary. TED-TP data can be accessed at 10.11888/Hydro.tpdc.271236. REA-ET data can be accessed at 10.5281/zenodo.4595941. ETM data can be accessed at 10.11888/RemoteSen.tpdc.272831.

## Code availability

Code necessary to replicate the Precipitation-to-TWSC model is available at 10.6084/m9.figshare.22769675. NSGA-II is provided by PlatEMO at https://github.com/BIMK/PlatEMO. The VIC model is a fully open-source model available at https://github.com/UW-Hydro/VIC.
