# Supplementary material for: Seasonal catchment memory of high mountain rivers in the Tibetan Plateau
Source: Nat Commun. 2023 Jun 1;14:3173. doi: 10.1038/s41467-023-38966-9 (PMC10235091; doi:10.1038/s41467-023-38966-9)
Supplement: Supplementary file 1 — Supplementary Information [file 41467_2023_38966_MOESM1_ESM.pdf]

Supplementary information for "Seasonal catchment memory of high mountain rivers in the Tibetan Plateau"

Haiting Gu[1,2], **Yue-Ping Xu**[1, 2], Li Liu[1,2], Jingkai Xie[1,2], Lu Wang[1,2], Suli Pan[1,2], Yuxue Guo[1,2]

1 Institute of Water Science and Engineering, Zhejiang University, Hangzhou 310058, China

2 College of Civil Engineering and Architecture, Zhejiang University, Hangzhou 310058, China

Supplementary Figures

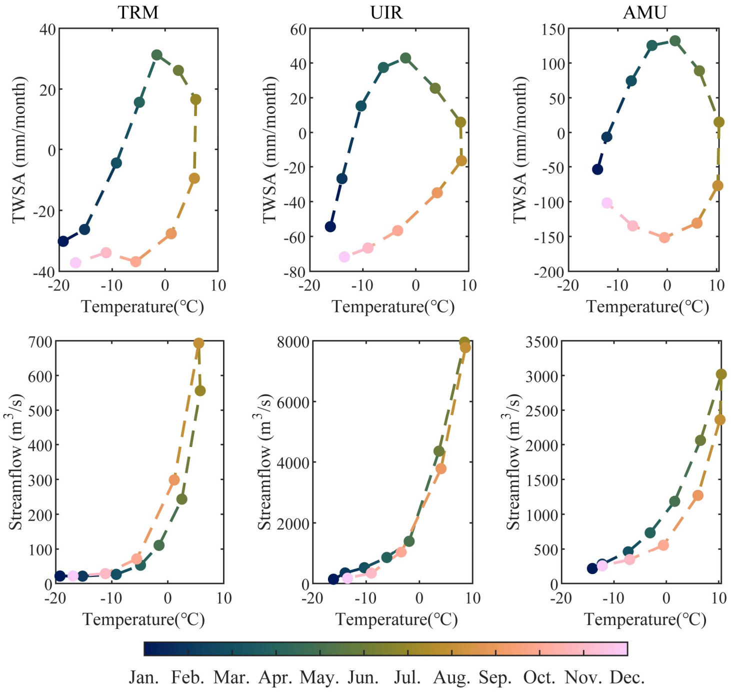

Supplementary Figure 1 Mean annual hysteresis plots between terrestrial water storage anomalies (TWSA), streamflow, and temperature. The hysteresis loops are plotted with monthly mean data from 2003 to 2018. Abbreviations TRM, UIR, and AMU stand for the upper Tarim River basin, the upper Indus River basin, and the upper Amu Darya basin, respectively.

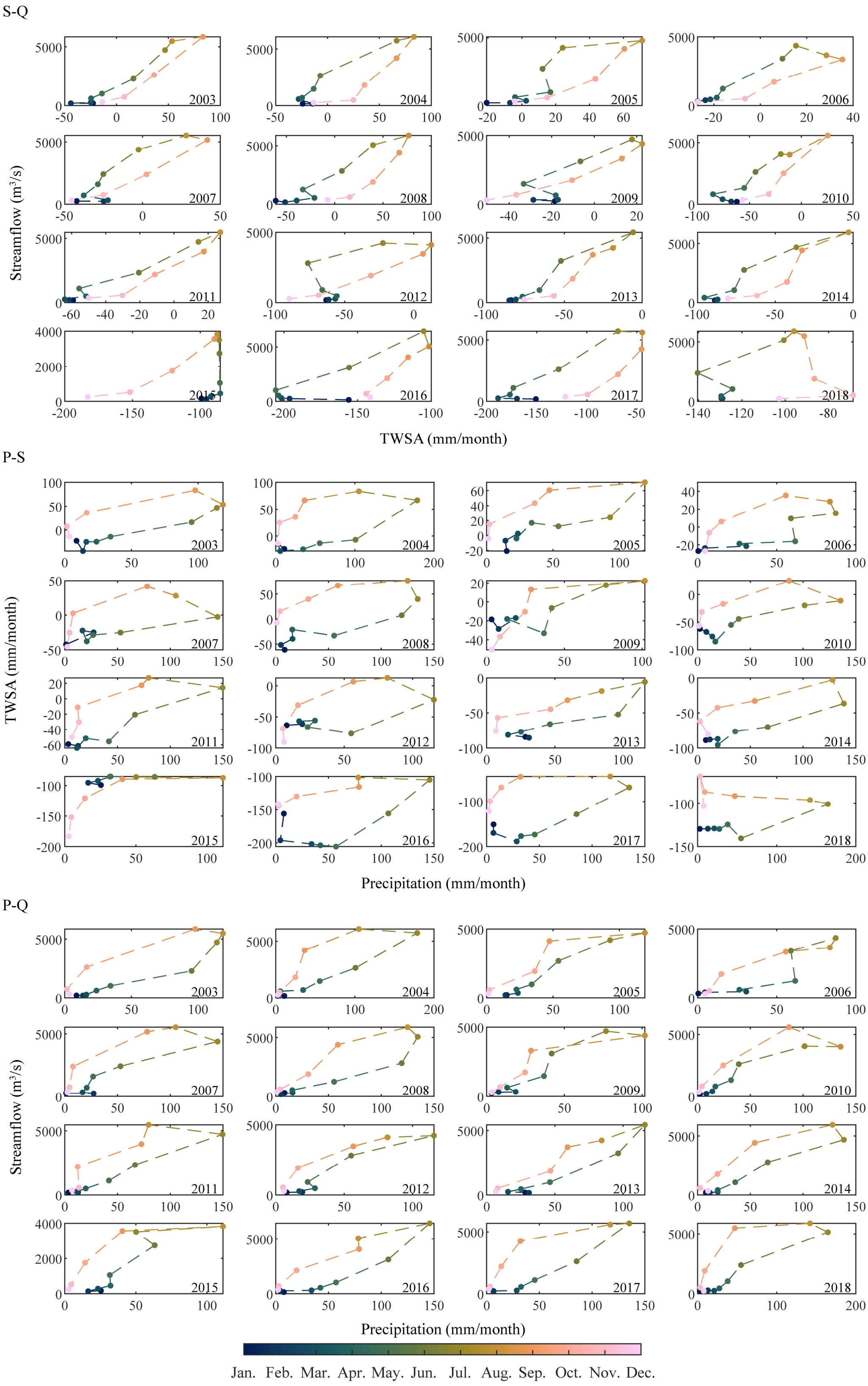

Supplementary Figure 2 Hysteresis plots between precipitation (P), streamflow (Q) and terrestrial water storage anomalies (TWSA, S) from 2003 to 2018 in the upper Brahmaputra River basin.

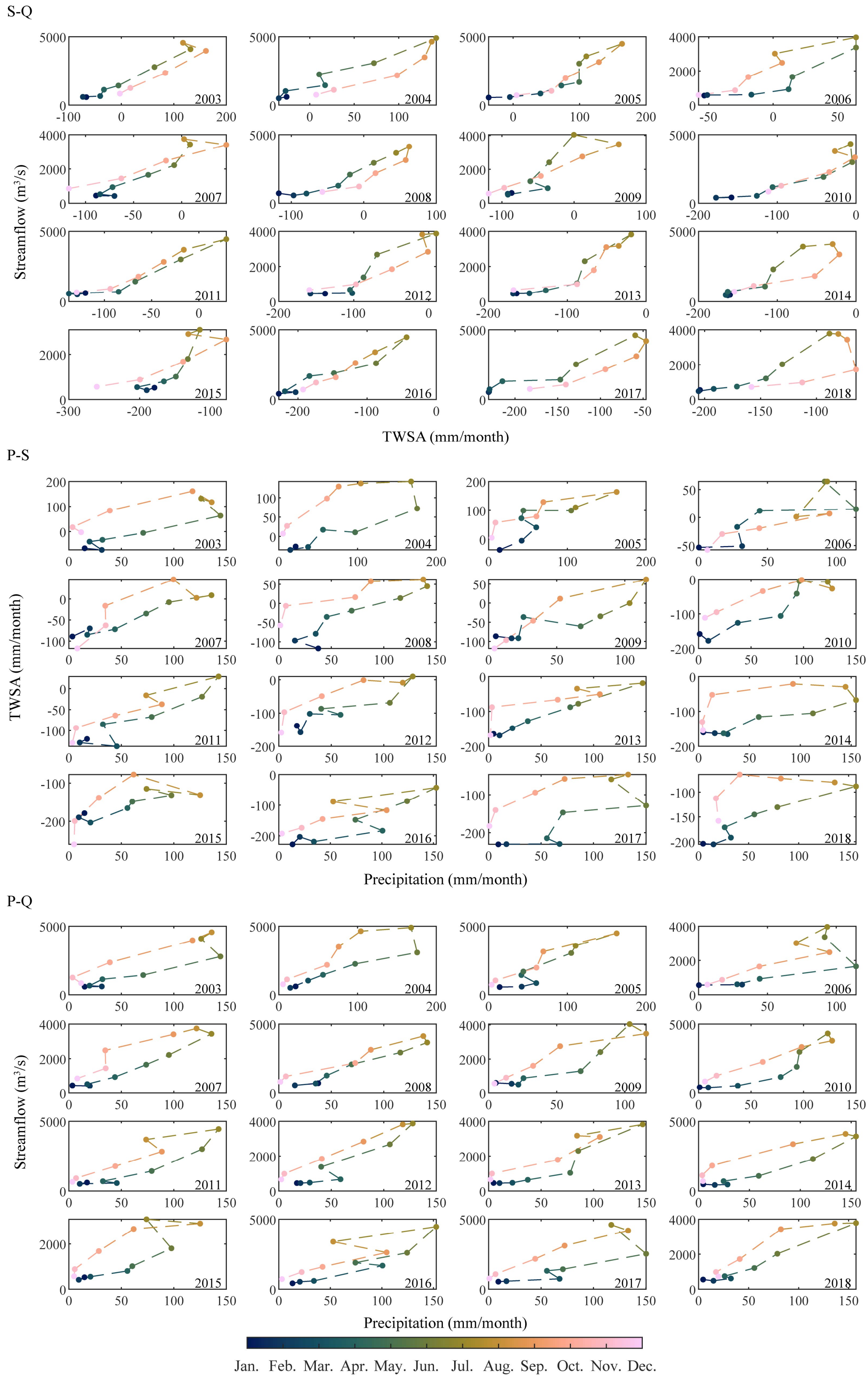

Supplementary Figure 3 Hysteresis plots between precipitation (P), streamflow (Q) and terrestrial water storage anomalies (TWSA, S) from 2003 to 2018 in the Salween River basin.

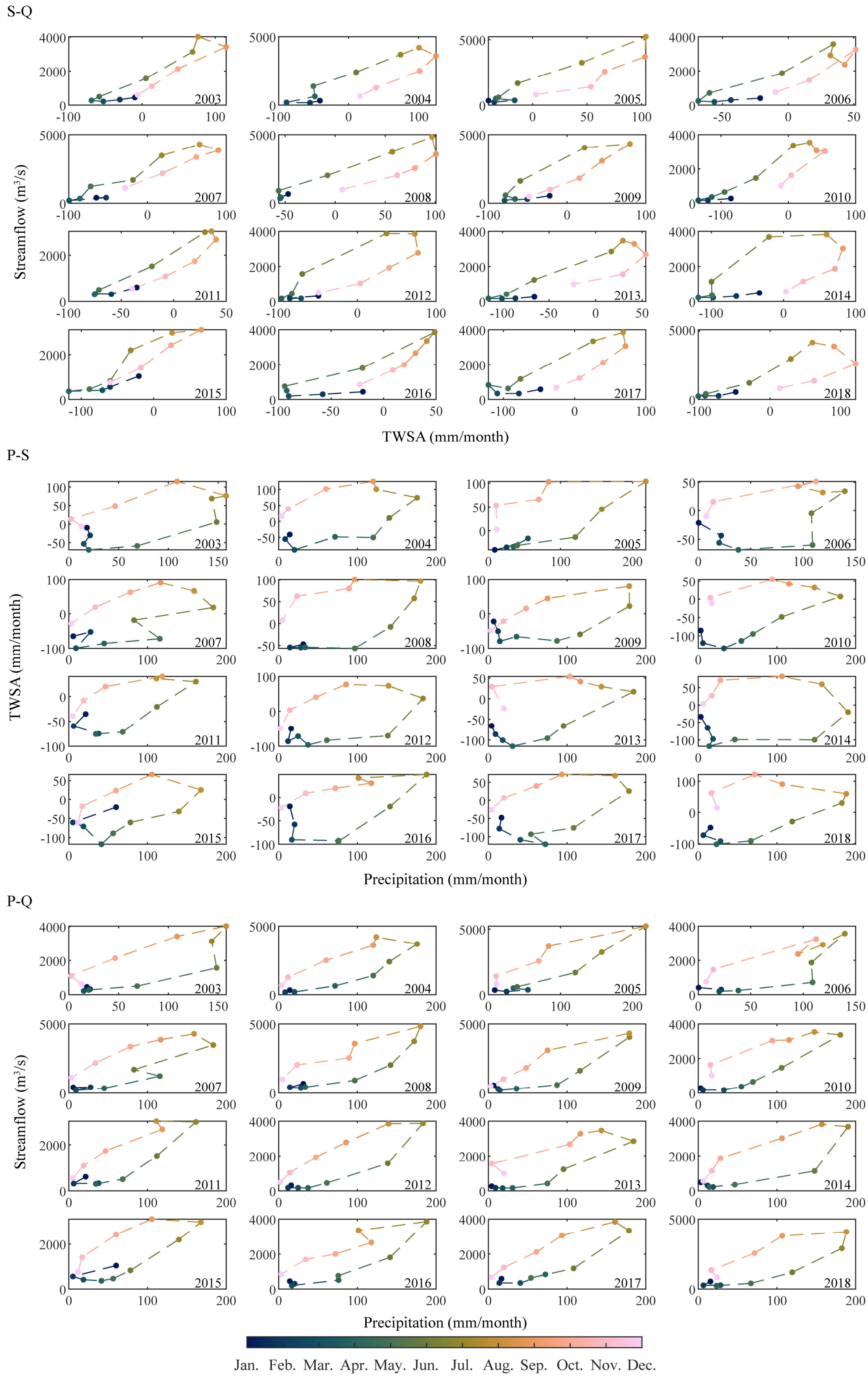

Supplementary Figure 4 Hysteresis plots between precipitation (P), streamflow (Q) and terrestrial water storage anomalies (TWSA, S) from 2003 to 2018 in the Lancang River basin.

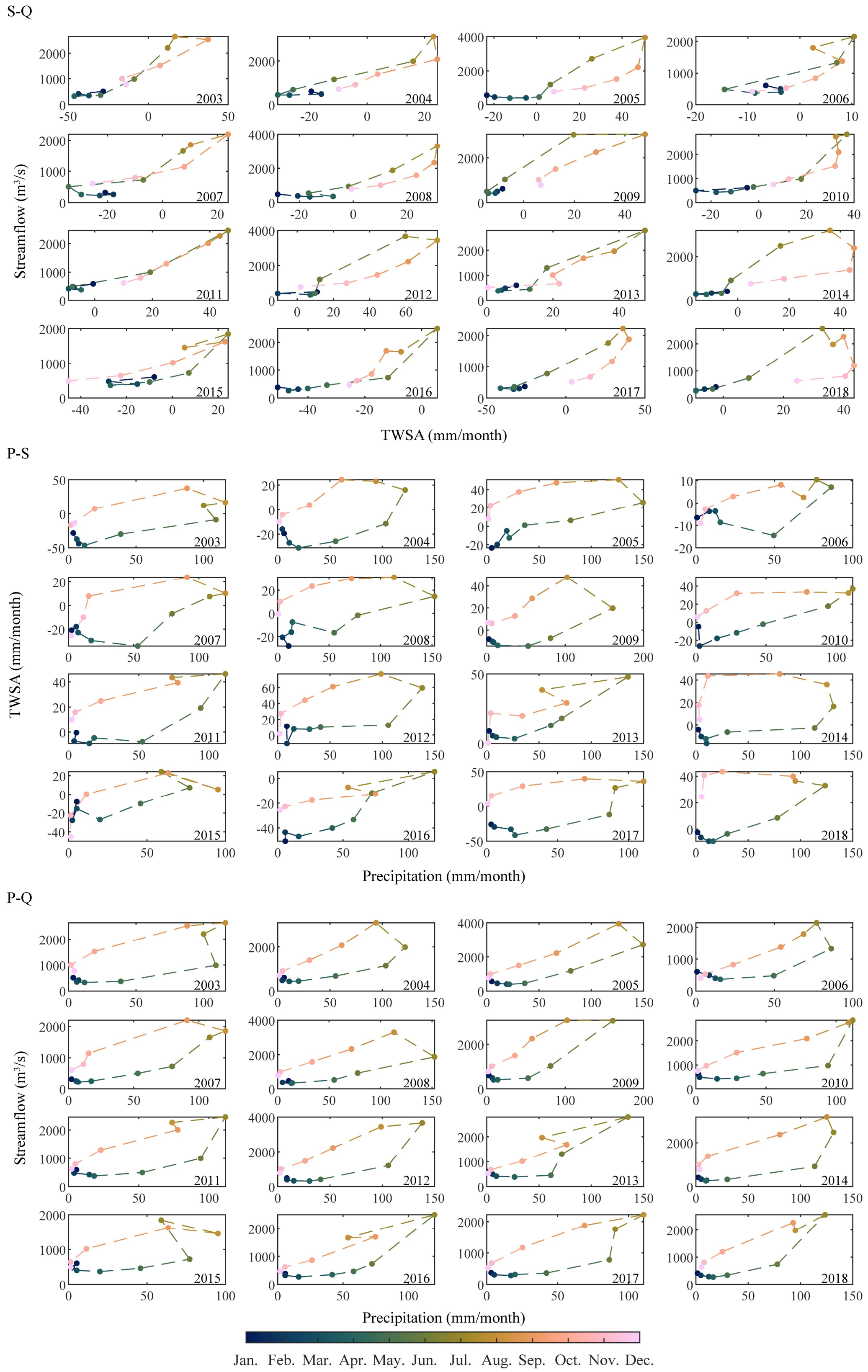

Supplementary Figure 5 Hysteresis plots between precipitation (P), streamflow (Q) and terrestrial water storage anomalies (TWSA, S) from 2003 to 2018 in the upper Yangtze River basin.

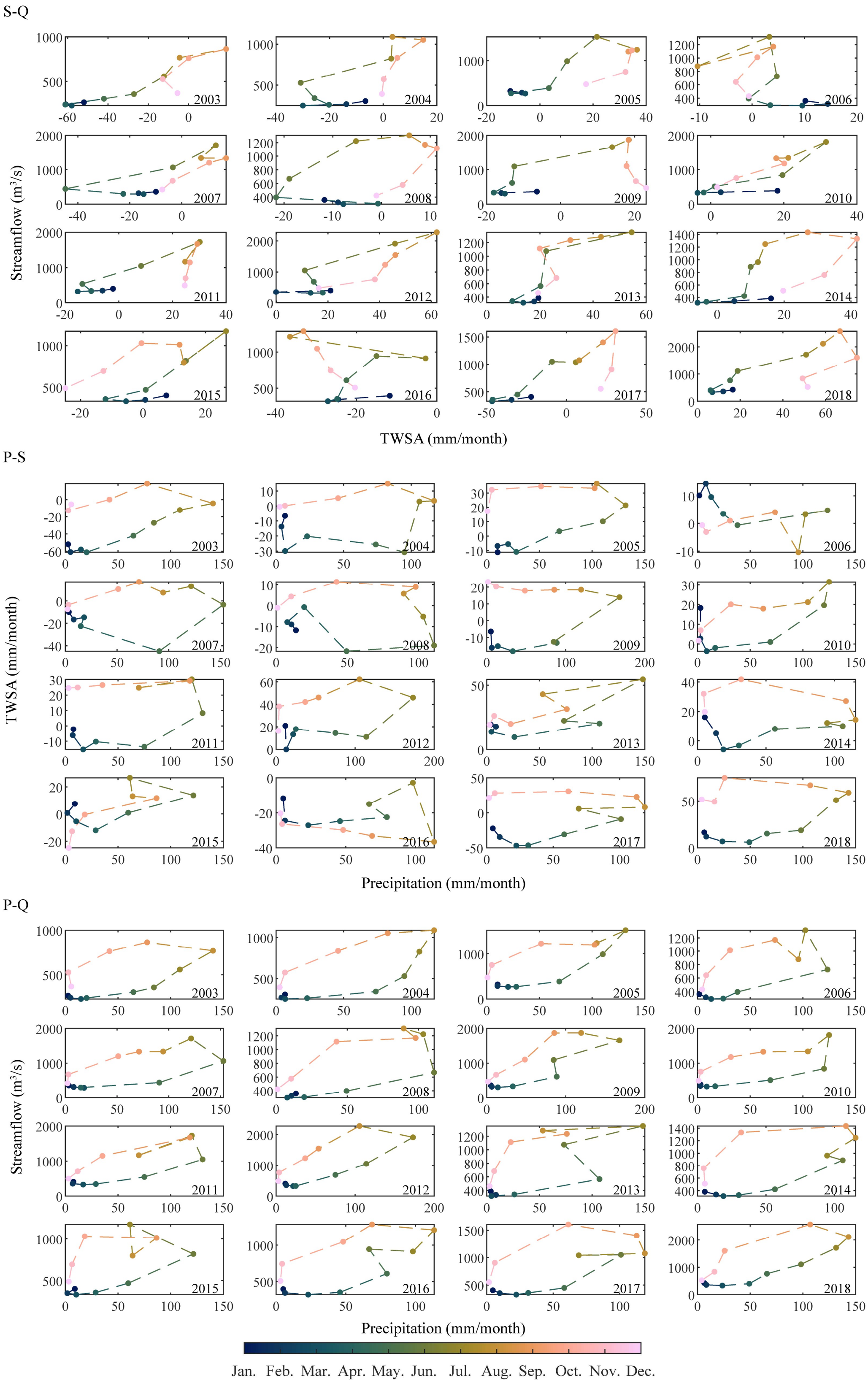

Supplementary Figure 6 Hysteresis plots between precipitation (P), streamflow (Q) and terrestrial water storage anomalies (TWSA, S) from 2003 to 2018 in the upper Yellow River basin.

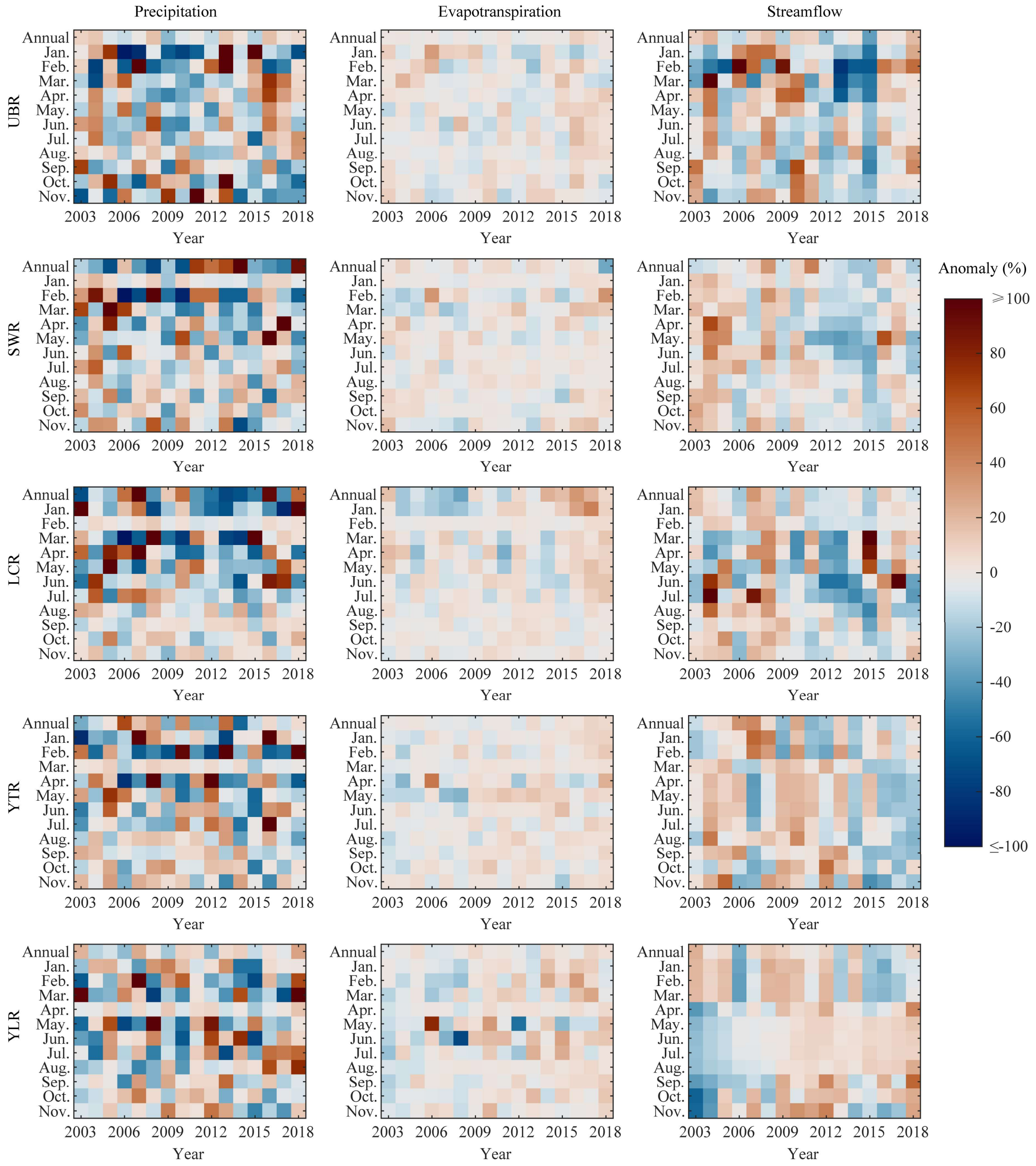

Supplementary Figure 7 Precipitation anomaly, evapotranspiration anomaly, and streamflow anomaly during the period 2003-2018. The average values are calculated with the data from 2003 to 2018. Abbreviations UBR, SWR, LCR, YTR, and YLR stand for the upper Brahmaputra River basin, the Salween River basin, the Lancang River basin, the upper Yangtze River basin, and the upper Yellow River basin, respectively.

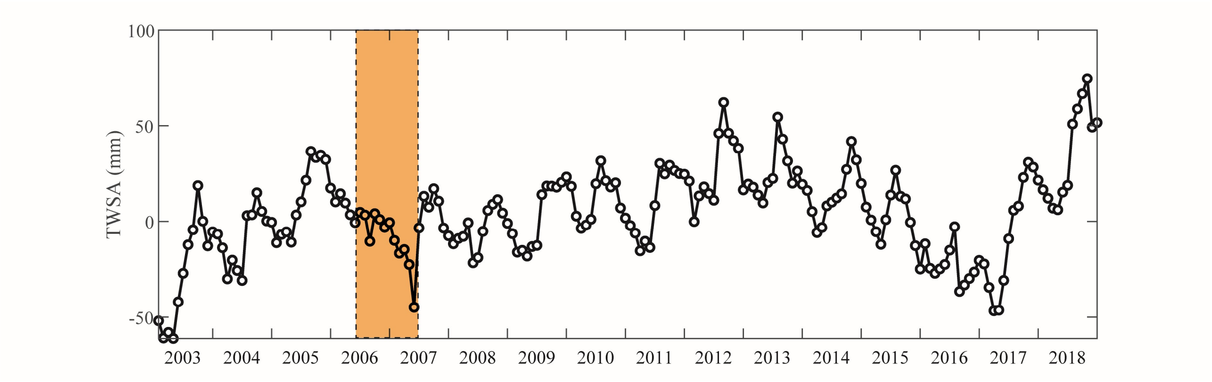

Supplementary Figure 8 GRACE-derived terrestrial water storage anomalies (TWSA) in the upper Yellow River basin from 2003 to 2018. The highlighted part is observed that TWSA in 2006 has a decrease from 10.1 mm in January to -9.9 mm in December, which continues into May of 2007.

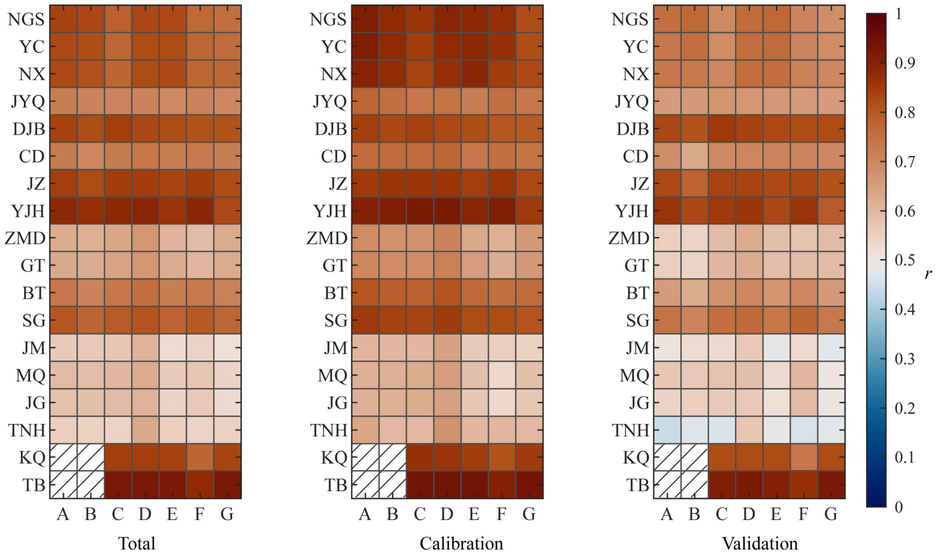

Supplementary Figure 9 Performance of the precipitation-to-TWSC model derived with seven precipitation products in 16 sub-basins for the total, calibration and validation periods. A to G represent seven precipitation products, i.e., the China Meteorological Forcing Dataset (CMFD), the China Gauge-based Daily Precipitation Analysis dataset (CGDPA), the Tropical Rainfall Measuring Mission 3b42v7 dataset (TRMM), the Integrated Multi-satellitE Retrievals for GPM Final Run Version 6 dataset (IMERG), the ECMWF Reanalysis v5 monthly averaged data (ERA5), the Climate Prediction Center Morphing Technique Climate Data Record (CMORPH) and the Precipitation Estimation from Remotely Sensed Information using Artificial Neural Networks - Climate Data Record (PERSIANN).

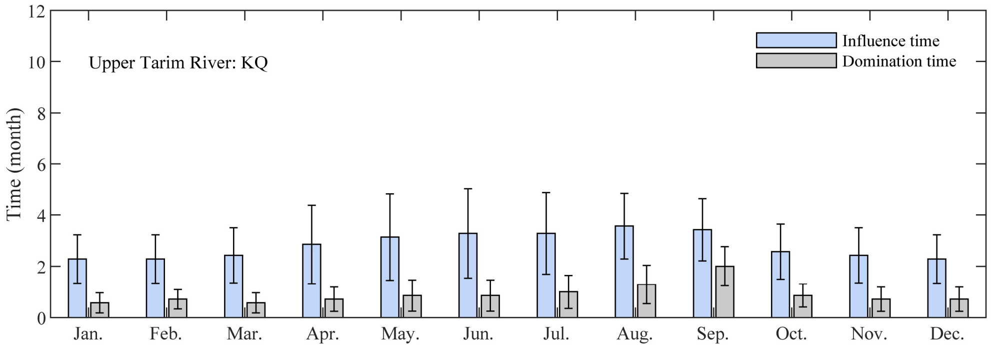

Supplementary Figure 10 Seasonal catchment memory duration in different months in the upper Tarim River basin. The influence time and domination time are the mean value of results from seven precipitation products. The error bar is the standard deviation of the influence/domination time from seven precipitation products, representing the uncertainty of the influence/domination time.

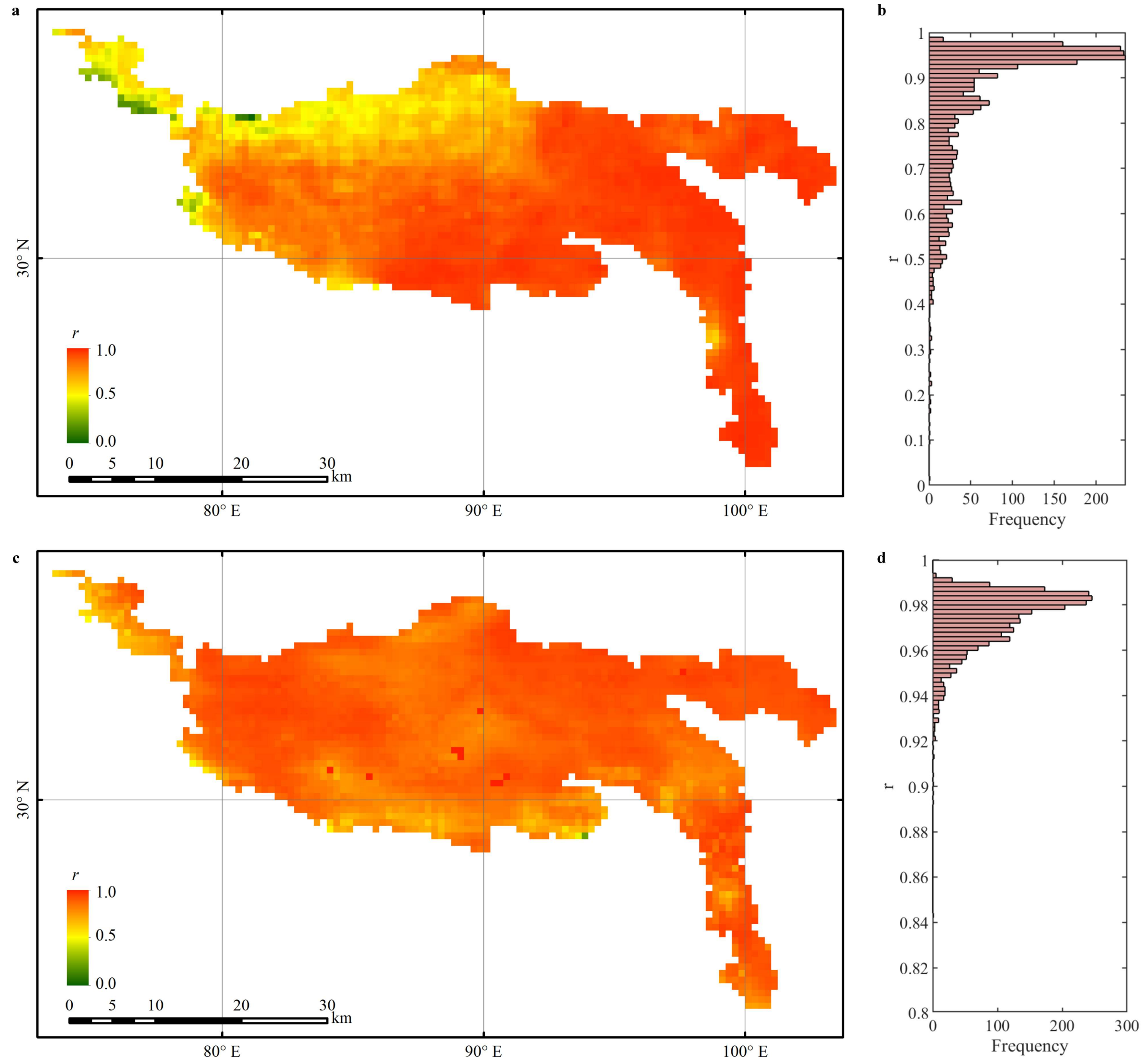

Supplementary Figure 11 Consistency analysis of model-driving precipitation and temperature data from different sources. **a** Spatial distribution of correlation coefficients (*r*) of precipitation between the China Meteorological Forcing Dataset (CMFD) and the Integrated Multi-satellitE Retrievals for GPM Final Run Version 6 dataset (IMERG). **b** Statistics of *r* between CMFD and IMERG precipitation. **c** Spatial distribution of *r* of temperature between CMFD and the Global Land Data Assimilation System dataset (GLDAS). **d** Statistics of *r* between CMFD and GLDAS temperature. The consistency of driving data is analyzed in the scale covered by different data sources, including the upper Brahmaputra River basin, the Salween River basin, the Lancang River basin, the upper Yangtze River basin, the upper Yellow River basin, the upper Tarim River basin and the Qiangtang Plateau.

Supplementary Tables

Supplementary Table 1 Stations used for calibration and validation in this study.

Basin	Station	Calibration period	Validation period	Area ($10^4 km^2$)	Q (m^3/s)	P (mm)	ET (mm)	Glacier area Ratio	ET source
UBR	NGS	2000-2008	2009-2015	11.2	540	419	367	1.22%	TDE-TP
	YC	2000-2008	2009-2015	15.6	1043	449	407	1.30%	TDE-TP
	NX*	2000-2008	2009-2015	19.6	1965	499	441	1.88%	TDE-TP
SWR	JYQ	2000-2006	2007-2010	7.6	865	608	481	1.79%	TDE-TP
	DJB*	2000-2006	2007-2015	11.7	1853	691	525	1.73%	TDE-TP
LCR	CD	2000-2005	2006-2010	5.5	469	579	491	0.36%	RAE-ET
	JZ	2000-2005	2006-2014	9.3	1002	617	553	0.37%	RAE-ET
	YJH*	2000-2005	2006-2008	14.3	1533	813	629	0.23%	RAE-ET
YTR	ZMD	2006-2011	2012-2015	13.9	499	444	360	0.77%	TDE-TP
	GT	2006-2011	2012-2015	15.5	646	458	368	0.72%	TDE-TP
	BT	2006-2011	2012-2015	18.9	991	478	406	0.64%	TDE-TP
	SG*	2006-2011	2012-2015	22.1	1302	498	443	0.58%	TDE-TP
YLR	JM	2007-2011	2012-2015	5.1	158	505	327	>0.01%	TDE-TP
	MQ	2007-2011	2012-2015	8.8	451	583	392	>0.01%	TDE-TP
	JG	2007-2011	2012-2015	10.2	546	589	402	>0.01%	TDE-TP
	TNH*	2000-2011	2012-2015	12.4	590	579	396	0.08%	TDE-TP
TRM	KQ*	2001-2007	2008-2011	4.7	229	270	222	9.62%	TDE-TP
UIR	TB*	2005-2013	2014-2018	17.3	2455	338	267	14.91%	ETM
AMU	NN*	2001-2010	2011-2016	11.7	1140	331	256	11.52%	ETM

Note: The annual sub-basin streamflow is calculated with the streamflow data during calibration and validation periods. The annual sub-basin precipitation and ET are calculated with the CMFD/IMERG and TDE-TP/ REA-ET/ETM, from 2003 to 2018, respectively. The Glacier area ratios are calculated from the Second Glacier Inventory of China (for UBR, SWR, LCR, YTR, YLR and TRM) and WestDC Global Glacier Dataset from National Cryosphere Desert Data Center (for UIR and AMU).

Abbreviations: Q, streamflow; P, precipitation; ET, evapotranspiration. UBR, the upper Brahmaputra River basin; SWR, the Salween River basin; LCR, the Lancang River basin; YTR, the upper Yangtze River basin; YLR, the upper Yellow River basin; TRM, the upper Tarim River basin; UIR, the upper Indus River basin; AMU, the upper Amu Darya basin. TDE-TP, monthly 0.01° terrestrial evapotranspiration datasets over the Tibetan Plateau from 2000 to 2018; REA-ET, the harmonized global land ET product with the reliability ensemble averaging method; ETM, the global ET dataset based on ETMonitor model.

Supplementary Table 2 Mann-Kendall z-statistics for TWSA, streamflow, precipitation and ET in the eight study basins.

Item	Period	UBR	SWR	LCR	YTR	YLR	TRM	UIR	AMU
TWSA	Annual	-4.45	-4.38	-2.30	1.22	1.49	-4.46	-4.37	-2.30
	MAM	-4.36	-4.46	-3.02	0.32	1.03	-4.37	-4.46	-3.02
	JJA	-4.28	-4.55	-2.75	1.04	1.76	-4.28	-4.55	-2.75
	SON	-4.46	-4.19	-0.77	1.13	1.67	-4.46	-4.19	-0.77
	DJF	-3.92	-4.37	-2.30	0.95	1.22	-3.92	-4.37	-2.30
Streamflow	Annual	-0.50	-2.12	-1.22	-1.49	1.94	-2.30	3.11	0.86
	MAM	-2.03	-1.85	-0.77	-1.76	3.29	-2.84	1.40	0.59
	JJA	0.77	-1.49	-2.12	-1.13	0.86	-2.21	2.93	1.04
	SON	0.32	-1.22	-0.59	-1.58	2.48	-2.12	1.85	0.86
	DJF	0.50	-0.77	0.68	-1.22	4.28	-2.21	-1.49	1.31
Precipitation	Annual	0.41	-1.22	-0.41	-1.40	0.14	0.41	-1.22	-0.41
	MAM	0.86	-0.23	-1.22	0.59	1.85	0.86	-0.23	-1.22
	JJA	0.9	-0.77	-0.05	-1.22	-0.86	0.59	-0.77	-0.05
	SON	-1.31	-0.86	0.05	-0.14	0.23	-1.31	-0.86	0.05
	DJF	0.32	-2.30	-0.68	-1.94	0.50	0.32	-2.30	-0.68
ET	Annual	0.77	-0.32	1.76	0.50	1.58	-3.11	1.04	0.00
	MAM	0.68	-0.23	0.23	0.05	2.12	-3.83	0.05	-0.95
	JJA	0.77	0.14	1.31	0.32	0.23	-1.76	1.58	0.14
	SON	0.14	0.77	3.29	0.05	0.68	-0.23	0.32	-0.95
	DJF	-1.13	-0.32	1.67	-1.22	1.04	0.05	-1.40	0.59

Note: A positive z-statistic means increasing trend, while a negative one means decreasing trend. Test statistic for a significant level of 5% is 1.96. When the statistic is larger than 1.96, the trend is significant. The streamflow and ET data are VIC-calculated.
Abbreviations: MAM, May to July; JJA, July to August; SON, September to November; DJF, December to next February.

Supplementary Table 3 Climate zone and land cover of sub-basins in the precipitation-dominated basins

Basin	Sub-basin	Climate zone						Land cover				
UBR	NGS	Cwb	Dwc	BSk	BWk	Dwd	-	G	-	-	-	-
		52%	23%	14%	5%	5%	-	98%	-	-	-	-
	YC	Cwb	Dwc	BSk	Dwd	-	-	G	-	-	-	-
		37%	23%	21%	14%	-	-	97%	-	-	-	-
	NX	Cwb	BSk	Dwc	BWk	Dwd	-	G	-	-	-	-
		34%	23%	20%	12%	11%	-	91%	-	-	-	-
SWR	JYQ	ETH	Dwd	Dwc	-	-	-	G	-	-	-	-
		42%	36%	22%	-	-	-	92%	-	-	-	-
	DJB	ETH	Dwd	Cwb	Dwc	Cfb	-	G	EN	OS	WG	-
		26%	23%	22%	18%	10%	-	77%	7%	7%	6%	-
LCR	CD	Dwc	Dwd	ETH	-	-	-	G	OS	-	-	-
		45%	38%	17%	-	-	-	90%	5%	-	-	-
	JZ	Dwc	Dwd	Cwb	ETH	Cfb	BSk	G	OS	EN	WG	-
		40%	22%	11%	10%	9%	7%	75%	8%	7%	7%	-
	YJH	Cwa	Dwc	Dwd	Cwb	Cfb	ETH	G	OS	WG	Cp	EN
		26%	26%	14%	13%	9%	6%	55%	12%	12%	9%	9%
YTR	ZMD	ETH	Dwd	BSk	-	-	-	G	-	-	-	-
		78%	11%	8%	-	-	-	99%	-	-	-	-
	GT	ETH	Dwc	Dwd	BSk	-	-	G	-	-	-	-
		72%	11%	10%	7%	-	-	98%	-	-	-	-
	BT	ETH	Dwc	BSk	Dwd	-	-	G	-	-	-	-
		59%	16%	11%	8%	-	-	93%	-	-	-	-
	SG	ETH	Dwc	BSk	Dwd	Cwb	-	G	EN	-	-	-
		50%	23%	10%	7%	7%	-	86%	5%	-	-	-
YLR	JM	ETH	Dwd	BSk	-	-	-	G	-	-	-	-
		69%	18%	13%	-	-	-	99%	-	-	-	-
	MQ	ETH	Dwd	BSk	-	-	-	G	-	-	-	-
		48%	41%	8%	-	-	-	96%	-	-	-	-
	JG	ETH	Dwd	BSk	-	-	-	G	-	-	-	-
		46%	44%	7%	-	-	-	95%	-	-	-	-
	TNH	Dwd	ETH	BSk	-	-	-	G	CS	-	-	-
		49%	43%	5%	-	-	-	93%	6%	-	-	-

Note: The table displays the climate zone and the type of land cover, whose proportion is greater than 5% in the sub-basins. The data of climate zone are from the updated world map of the Köppen-Geiger climate classification[1]. The land cover data are from the WestDC land cover dataset. The land cover classification is referred to University of Maryland (UMD) land cover classification[2].

Abbreviations: The abbreviations in climate zone are referred to the study of Peel et al.[1]. Cp, Croplands; CS, Closed Shrublands; EN, Evergreen Needleleaf Forests; G, Grasslands; OS, Open Shrublands; WG, Wooded grasslands. UBR, the upper Brahmaputra River basin; SWR, the Salween River basin; LCR, the Lancang River basin; YTR, the upper Yangtze River basin; YLR, the upper Yellow River basin.

Supplementary Table 4 Soil type of sub-basins in the precipitation-dominated basins

Basin	Subbasin	Topsoil				Subsoil				
UBR	NGS	C	SaL	L	LS	SaL	L	C	SCL	-
		35%	25%	25%	9%	33%	23%	19%	18%	-
	YC	C	SaL	L	LS	SaL	L	C	SCL	LS
		32%	29%	24%	9%	35%	22%	20%	14%	5%
	NX	C	L	SaL	LS	SaL	L	C	SCL	LS
		31%	28%	27%	10%	32%	26%	21%	11%	7%
SWR	JYQ	SaL	L	-	-	SaL	L	-	-	-
		70%	30%	-	-	71%	25%	-	-	-
	DJB	SaL	L	-	-	SaL	L	CL	-	-
		58%	39%	-	-	59%	26%	14%	-	-
LCR	CD	SaL	L	SiL	-	SaL	L	CL	-	-
		66%	28%	6%	-	66%	27%	7%	-	-
	JZ	SaL	L	-	-	SaL	L	CL	-	-
		52%	44%	-	-	52%	30%	18%	-	-
	YJH	L	SaL	C	SCL	SaL	L	CL	C	-
		43%	33%	15%	5%	33%	27%	19%	19%	-
YTR	ZMD	SaL	L	-	-	SaL	L	CL	-	-
		74%	23%	-	-	74%	17%	7%	-	-
	GT	SaL	L	-	-	SaL	L	CL	-	-
		73%	23%	-	-	73%	18%	7%	-	-
	BT	SaL	L	-	-	SaL	L	CL	-	-
		65%	30%	-	-	65%	23%	10%	-	-
	SG	SaL	L	-	-	SaL	L	CL	-	-
		59%	37%	-	-	59%	24%	15%	-	-
YLR	JM	SaL	L	-	-	SaL	CL	-	-	-
		88%	10%	-	-	88%	8%	-	-	-
	MQ	SaL	L	-	-	SaL	L	CL	-	-
		65%	33%	-	-	65%	23%	9%	-	-
	JG	SaL	L	-	-	SaL	L	CL	-	-
		62%	37%	-	-	62%	27%	9%	-	-
	TNH	SaL	L	-	-	SaL	L	CL	-	-
		65%	33%	-	-	65%	25%	7%	-	-

Note: The table displays the soil type whose proportion is greater than 5% in the sub-basins. The soil data is from the HWSD soil dataset and the soil classification is referred to U.S. Department of Agriculture (USDA) classification[3].

Abbreviations: C, Clay; CL, Clay loam; L, Loam; LS, Loamy Sand; SaL, Sandy Loam; SL, Silt Loam; SCL, Sandy Clay Loam. UBR, the upper Brahmaputra River basin; SWR, the Salween River basin; LCR, the Lancang River basin; YTR, the upper Yangtze River basin; YLR, the upper Yellow River basin.

Supplementary Table 5 Basic information of seven precipitation datasets

Dataset	Spatial resolution	Temporal resolution	Data length	Data available
CMFD	0.1°	1 day	1979-2018	http://data.tpdc.ac.cn/en/data/8028b944-daaa-4511-8769-965612652c49/
CGDPA	0.25°	1 day	1971-2018	http://data.cma.cn/data/cdcdetail/dataCode/SEVP_CLI_CHN_PRE_DAY_GRID_0.25.html
TRMM	0.25°	1 day	1998-2019	https://disc.gsfc.nasa.gov/datasets/TRMM_3B42_Daily_7/summary
IMERG	0.1°	1 day	2000-present	https://gpm.nasa.gov/data/imerg
ERA5	0.1°	1 month	1950-present	https://cds.climate.copernicus.eu/cdsapp#!/dataset/reanalysis-era5-land-monthly-means?tab=form
CMORPH	0.25°	1 day	1998-present	https://www.ncei.noaa.gov/products/climate-data-records/precipitation-cmorph
PERSIANN	0.25°	1 day	1983-present	https://www.ncei.noaa.gov/products/climate-data-records/precipitation-cmorph

Abbreviations: CMFD, the China Meteorological Forcing Dataset; CGDPA, the China Gauge-based Daily Precipitation Analysis dataset; TRMM, the Tropical Rainfall Measuring Mission 3b42v7 dataset; IMERG, the Integrated Multi-satellitE Retrievals for GPM Final Run Version 6 dataset; ERA5, the ECMWF Reanalysis v5 monthly averaged data; CMORPH, the Climate Prediction Center Morphing Technique Climate Data Record; PERSIANN, the Precipitation Estimation from Remotely Sensed Information using Artificial Neural Networks - Climate Data Record.

Supplementary References

1. Peel, M. C., Finlayson, B. L. & McMahon, T. A. Updated world map of the Köppen-Geiger climate classification. *Hydrol. Earth Syst. Sci.* **11**, 1633-1644 (2007).
2. Hansen, M. C., DeFries, R. S., Townshend, J. R. & Sohlberg, R. Global land cover classification at 1 km spatial resolution using a classification tree approach. *Int. J. Remote Sens.* **21**, 1331-1364 (2000).
3. USDA, N. Keys to soil taxonomy. *Soil Survey Staff, Washington*, (2010).
